# Comparison of video laryngoscopy with direct laryngoscopy for intubation success in critically ill patients: a systematic review and Bayesian network meta-analysis

**DOI:** 10.3389/fmed.2023.1193514

**Published:** 2023-06-09

**Authors:** Jae Guk Kim, Chiwon Ahn, Wonhee Kim, Tae-Ho Lim, Bo-Hyong Jang, Youngsuk Cho, Hyungoo Shin, Heekyung Lee, Juncheol Lee, Kyu-Sun Choi, Min Kyun Na, Sae Min Kwon

**Affiliations:** ^1^Department of Emergency Medicine, Kangnam Sacred Heart Hospital, Hallym University College of Medicine, Seoul, Republic of Korea; ^2^Department of Emergency Medicine, College of Medicine, Chung-Ang University, Seoul, Republic of Korea; ^3^Department of Emergency Medicine, Hanyang University College of Medicine, Seoul, Republic of Korea; ^4^Department of Preventive Medicine, College of Korean Medicine, Kyung Hee University, Seoul, Republic of Korea; ^5^Department of Emergency Medicine, Kangdong Sacred Heart Hospital, Hallym University College of Medicine, Seoul, Republic of Korea; ^6^Department of Neurosurgery, Hanyang University College of Medicine, Seoul, Republic of Korea; ^7^Department of Neurosurgery, Dongsan Medical Center, Keimyung University School of Medicine, Daegu, Republic of Korea

**Keywords:** laryngoscopy, intubation, critical care, randomized controlled trial, network meta-analysis

## Abstract

**Introduction:**

This review compares the efficacy of video laryngoscopy (VL) with direct laryngoscopy (DL) for successful tracheal intubation in critically ill or emergency-care patients.

**Methods:**

We searched the MEDLINE, Embase, and Cochrane Library databases for randomized controlled trials (RCTs) that compared one or more video laryngoscopes to DL. Sensitivity analysis, subgroup analysis, and network meta-analysis were used to investigate factors potentially influencing the efficacy of VL. The primary outcome was the success rate of first-attempt intubation.

**Results:**

This meta-analysis included 4244 patients from 22 RCTs. After sensitivity analysis, the pooled analysis revealed no significant difference in the success rate between VL and DL (VL vs. DL, 77.3% vs. 75.3%, respectively; OR, 1.36; 95% CI, 0.84–2.20; I^2^ = 80%; low-quality evidence). However, based on a moderate certainty of evidence, VL outperformed DL in the subgroup analyses of intubation associated with difficult airways, inexperienced practitioners, or in-hospital settings. In the network meta-analysis comparing VL blade types, nonchanneled angular VL provided the best outcomes. The nonchanneled Macintosh video laryngoscope ranked second, and DL ranked third. Channeled VL was associated with the worst treatment outcomes.

**Discussion:**

This pooled analysis found, with a low certainty of evidence, that VL does not improve intubation success relative to DL. Channeled VL had low efficacy in terms of intubation success compared with nonchanneled VL and DL.

**Systematic review registration:**

https://www.crd.york.ac.uk/prospero/display_record.php?RecordID=285702, identifier: CRD42021285702.

## 1. Introduction

Endotracheal intubation (ETI) is important in life-threatening situations involving hypoxia or unconsciousness, including in the management of critically ill or emergency-care patients who require airway protection (1, 2). The Macintosh laryngoscope for direct laryngoscopy (DL) has been the preferred intubation approach for a half-century, and it is frequently used in both in-hospital and prehospital settings (3, 4). However, ETI success rates decrease in association with difficult airways or emergency circumstances (5–8), and intubation failure owing to the placement of the endotracheal tube into the esophagus, for example, can worsen the patient's hypoxia and result in brain death and, eventually, death (9). Therefore, attempts to increase ETI success rates and decrease complications include the use of appropriate sedatives and neuromuscular blockers, skilled and experienced personnel managing difficult airways, simulation-based training, and care bundles (10–12). Nonetheless, ETI failure may occur because of several influencing factors and unpredictable circumstances.

Video laryngoscopy (VL) can be used in clinical practice as an alternative to DL for ETI, wherein indirect assessment of the glottal structure is possible using a small camera mounted to the laryngoscope blade tip, as has been investigated in several studies of VL outcomes and comparisons of VL vs. DL ETI success rates. However, studies investigating various VL devices or different ETI settings, such as prehospital (13–16), emergency rooms (2, 17, 18), intensive care units (3, 10), or operating rooms (19, 20), have generated conflicting findings, especially comparative trials of VL vs. DL for critically ill and emergency-care patients that have contrasted with the findings associated with planned ETI in the operating room (10, 21–23). A previous meta-analysis of several cohort studies and three randomized trials determined considerably higher ETI success rates associated with VL (24), whereas a subsequent meta-analysis of only randomized trials found no significant difference in ETI success between VL and DL (1). The substantial heterogeneity revealed by the above-mentioned review needs attention, as multiple devices were analyzed using one-arm forced integration for the meta-analysis.

Lee et al. used a network meta-analysis (NMA) of various VL devices in patients scheduled to undergo ETI for surgery to identify the most effective devices; however, the comparison of too many devices made it difficult for clinicians to determine the optimal devices (25). Thus, a more comprehensive multi-arm analysis with clustering of similar VL devices may be required to overcome this issue.

Accordingly, in this review, VL devices were categorized according to the laryngoscope blade, and an NMA was conducted to compare VL with DL efficacy in ETI among critically ill or emergency-care patients. The meta-analysis investigated factors potentially influencing VL efficacy.

## 2. Methods

### 2.1. Protocol and registration

This review followed the PRISMA (Preferred Reporting Items for Systematic Reviews and Meta-Analyses) statement for reporting NMAs (26). The review protocol was registered with PROSPERO (CRD42021285702 available at: https://www.crd.york.ac.uk/prospero/display_record.php?RecordID=285702).

### 2.2. Eligibility criteria

Randomized controlled trials (RCTs) were eligible for inclusion if they compared one or more VL devices with DL or compared two or more VL devices without DL for the oral insertion of single-lumen endotracheal tubes in emergency-care or critically ill adult patients. The adequacy of ETI for emergency-care patients was evaluated in both the prehospital and in-hospital settings. In intensive care units, ETI was deemed to have been conducted on critically ill patients. Case reports, reviews, preprints, conference abstracts, observational studies, pediatric patients, cadaveric models, and manikin models were excluded, as were surgical patients requiring ETI for general anesthesia or those requiring urgent surgical airways, supraglottic airways, double-lumen tube installation, or nasal intubation.

### 2.3. Information sources and search strategy

An electronic search of the MEDLINE, Embase, and Cochrane Library databases yielded relevant literature without language restrictions, including articles published until October 31, 2022. Additional publications were identified by reviewing the reference lists of the identified papers and relevant previously published reviews. The full search strategy is provided in Supplementary Table S1.

### 2.4. Article selection and data collection

Title and abstract screening were carried out independently by two investigators (CA and JGK). The full texts of all potentially relevant citations were reviewed for eligibility. Articles were included in the review if they fulfilled the eligibility criteria and had data for at least one outcome of interest. Non-English papers deemed possibly relevant were evaluated for inclusion if the full text could be translated. Any disagreements were resolved through discussion. The same two investigators also independently extracted data.

### 2.5. Outcome measures

The primary outcome was first-attempt ETI success. Data on eligibility criteria, sample size, baseline characteristics of study participants, and devices evaluated were retrieved.

### 2.6. Assessment of risk of bias

The risk of bias within the included studies was assessed—using RoB 2 (Risk of Bias, version 2, Cochrane, London, UK) (27)—in terms of the following categories: “risk of bias arising from the randomization process,” “risk of bias due to deviations from the intended interventions,” “risk of bias due to missing outcome data,” “risk of bias in measurement of the outcome,” and “risk of bias in selection of the reported result,” with each subcategory rated as “yes,” “probably yes,” “no,” “probably no,” or “no information.” Based on the overall quality rating standards stated in RoB 2, the risk of bias was classified as “low,” “high,” or “some concerns.” Disagreements, if any, were resolved through discussion. Publication bias across individual articles was examined using funnel plots and Egger's regression test (28).

### 2.7. Reporting guidelines and certainty of evidence

The modified GRADE (Grades of Recommendation, Assessment, Development, and Evaluation) tool for meta-analyses was used to assess the quality of evidence (29). The following quality levels were assigned to the results: (1) high quality—further research is very unlikely to change the confidence in the estimated effect; (2) moderate quality—further research is likely to have an important impact on the confidence in the estimated effect and may change the estimate; (3) low quality—further research is very likely to have an important impact on the confidence in the estimated effect and is likely to change the estimate; and (4) very low quality, where any estimated effect is highly uncertain. We then used GRADE software (Evidence Prime, Hamilton, ON, Canada) to create a GRADE-evidence profile table to assess these outcomes as high, moderate, low, or very low quality.

### 2.8. Statistical analysis

Odds ratios (ORs) with 95% confidence intervals (CIs) were estimated for dichotomous outcomes, and statistically nonsignificant differences were indicated by ORs with 95% CIs that included 1. For subgroup analysis, continuous variables were converted to dummy variables using the 50% standard as follows: <50% vs. ≥50%. In cases of statistical heterogeneity (I^2^ ≥40%) or clinical heterogeneity, sensitivity analysis was performed to investigate potential sources of heterogeneity by sequentially eliminating individual articles using the Baujat plot (30). After excluding an outlier article from the sensitivity analysis, subgroup analyses for the primary outcome were performed based on the following potentially heterogeneous factors: (a) study design: single-center vs. multicenter; (b) study setting: prehospital vs. in-hospital (intensive care unit and emergency room); (c) country: non-Asian vs. Asian; (d) difficult airway proportion: <50% vs. ≥50%; (e) intubators' experience: inexperienced (nonphysician) vs. experienced (physician); (f) rapid sequence intubation: yes vs. no; and (g) the proportion of intubation during cardiopulmonary resuscitation (CPR): <50% vs. ≥50%. The experienced group for ETI was classified by the intubators' experience based on the standard criterion of physicians with sufficient ETI experience. The inexperienced group included students, paramedics, nurses, residents, and fellow trainees. Supplementary Table S2 contains more information on these factors; articles with missing data were excluded from the subgroup analysis. Meta-regression was conducted for two potentially heterogeneous factors: study recruitment start dates and sample sizes.

In the NMA of VL devices, DL with a Macintosh blade was designated as the control, and VL devices were classified into three categories according to their blades: nonchanneled Macintosh devices, nonchanneled angular devices, and channeled devices.

The reference management software Endnote 20 (Clarivate Analytics, Philadelphia, PA, USA) was used to organize all identified articles from the literature search. Meta-analyses, including the risk of bias assessment, sensitivity and subgroup analyses, and meta-regression, were performed for the comparison of VL and DL using R, version 4.2.1 (R Foundation for Statistical Computing, Vienna, Austria) and the following R packages: *meta, metafor*, and *rmeta*. A Bayesian NMA was performed using the *gemtc* R package (31). A random-effects model was used when impact size was pooled. The deviance information criteria and I^2^ heterogeneity were used to assess the inconsistency of the Bayesian NMA model (32). The best NMA model was obtained by minimizing deviance information criteria and I^2^ using Markov chain Monte Carlo simulation. The rank probability was used to rank each device's effectiveness and select the best device for an outcome. Rank probabilities range from 0 to 1; the closer an intervention's rank probability is to 1, the more likely it ranks first among available treatment options (33).

## 3. Results

### 3.1. Article selection and characteristics

**Figure 1:**
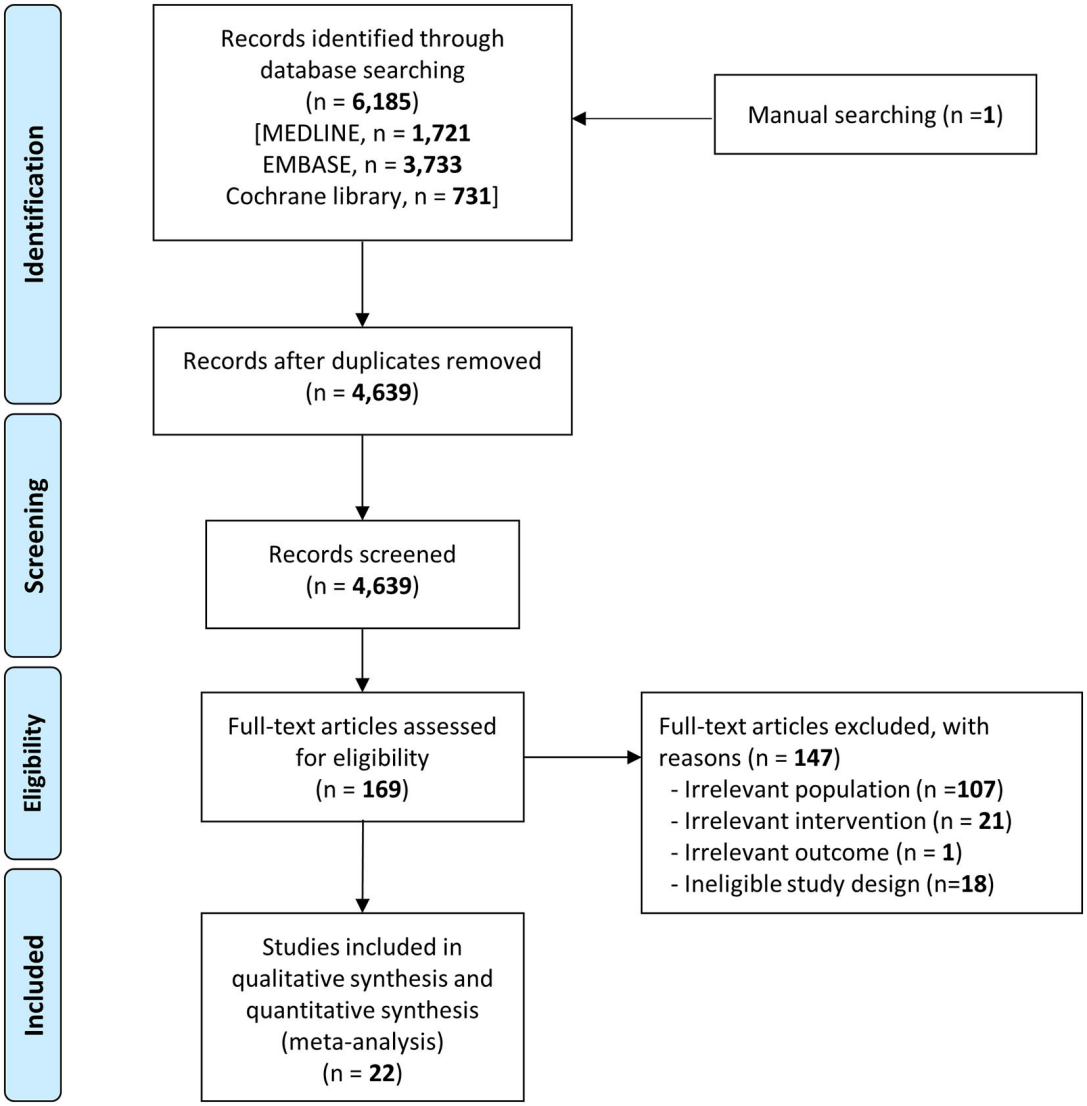
PRISMA flow chart depicting the disposition and selection of articles included in this systematic review and meta-analysis.

In total, 4,639 articles were included after eliminating duplicates. After the titles and abstracts were reviewed, a total of 169 papers were included for full-text review, after the exclusion of documents that did not meet the objectives of this review (Supplementary Table S3). The qualitative synthesis included 22 relevant articles (Figure 1) (2, 3, 10, 13, 15–18, 23, 34–46).

Table 1 shows the characteristics of the included articles comparing VL with DL, published from 2011 through 2021, with sample sizes ranging from 40 to 623 participants. Of the 22 studies included in this analysis, 4 were multicenter RCTs, while the others were single-center studies. Six studies were conducted in the prehospital setting, while the remaining 16 were carried out in the intensive care unit or emergency department, representing an in-hospital setting. ETI was performed by experienced operators in 11 of the studies, while ETI was mainly conducted by inexperienced operators in the remaining 11 studies. Sixteen studies used rapid sequence intubation with sedatives or narcotics and neuromuscular blockades during the intubation process. The following nine VL devices were evaluated in this review: Airtraq (Prodol, Vizcaya, Spain), Airwayscope (Nihon Kohden, Tokyo, Japan), C-MAC (Karl Storz, Tuttlingen, Germany), Glidescope (Verathon, Bothell, WA, USA), King Vision (Ambu, Copenhagen, Denmark), McGrath (Medtronic, Dublin, Ireland), Olympus Video Bronchoscope (Olympus, Tokyo, Japan), UEScope (UE Medical Corp., Zhejiang, China), and VivaSight (ETView Ltd., Misgav, Israel) (Supplementary Figure S1).

**Table 1:** Characteristics of the studies included in the review and meta-analysis.

**References**	**Study design**	**Setting**	**Country**	**Recruitment period**	**Patients, N**	**Laryngoscope** **(blade)**	**Difficult airway**	**Intubators' experience**	**Sedatives in RSI**	**Muscle relaxants in RSI**	**Intubation during CPR**
Trimmel et al. (34)	sRCT	Prehospital	Austria	2008–2009	212	Airtraq (C) vs. DL	<50%	Experienced	Used	Used	<50%
Griesdale et al. (35)	sRCT	ICU	Canada	2009–2011	40	Glidescope (A) vs. DL	<50%	Inexperienced	Used	Used	<50%
Yeatts et al. (36)	sRCT	ER	US	2008–2010	623	Glidescope (A) vs. DL	Unknown	Inexperienced	Used	Used	<50%
Arima et al. (37)	sRCT	Prehospital	Japan	2012–2013	118	Airwayscope (C) vs. DL	<50%	Experienced	Not used	Not used	≥50%
Ahmadi et al. (38)	sRCT	ER	Iran	2011	97	Glidescope (A) vs. DL	≥50%	Inexperienced	Unknown	Unknown	<50%
Silverberg et al. (3)	sRCT	ICU	US	2012–2013	117	Glidescope (A) vs. DL	<50%	Inexperienced	Used	Used	<50%
Driver et al. (18)	sRCT	ER	US	2011–2013	198	C-MAC (M) vs. DL	<50%	Inexperienced	Used	Used	<50%
Goksu et al. (23)	sRCT	ER	Turkey	2013–2014	150	C-MAC (M) vs. DL	Unknown	Inexperienced	Used	Unknown	<50%
Janz et al. (39)^*^	sRCT	ICU	US	2014–2015	150	McGrath (M), Glidescope (A), and Olympus bronchoscope^**^ vs. DL	<50%	Inexperienced	Used	Used	<50%
Kim et al. (17)	sRCT	ER	South Korea	2011–2013	140	Glidescope (A) vs. DL	Unknown	Experienced	Not used	Not used	≥50%
Sulser et al. (2)	sRCT	ER	Switzerland	2014–2015	147	C-MAC (M) vs. DL	<50%	Experienced	Used	Used	<50%
Trimmel et al. (15)	mRCT	Prehospital	Austria Norway	2011–2012	326	Glidescope (A) vs. DL	<50%	Experienced	Used	Used	≥50%
Ducharme et al. (40)	mRCT	Prehospital	US	2014–2016	82	KingVision (C) vs. DL	<50%	Inexperienced	Not used	Not used	≥50%
Lascarrou et al. (10)	mRCT	ICU	France	2015–2016	365	McGrath (M) vs. DL	<50%	Inexperienced	Used	Used	<50%
Abdelgalel et al. (41)	sRCT	ICU	Egypt	2016–2017	120	Airtraq (C) vs. Glidescope (A) vs. DL	Unknown	Experienced	Used	Used	<50%
Gao et al. (42)	sRCT	ICU	China	2014–2016	163	UEScope (M) vs. DL	<50%	Experienced	Used	Not used	<50%
Grensemann et al. (43)^*^	sRCT	ICU	Germany	2016–2018	53	VivaSight^***^ vs. DL	Unknown	Experienced	Used	Used	<50%
Kreutziger et al. (13)	mRCT	Prehospital	Austria	2017–2018	514	McGrath (M) vs. DL	<50%	Experienced	Used	Used	≥50%
Dey et al. (44)	sRCT	ICU	India	2017–2018	218	C-MAC (M) vs. DL	Unknown	Experienced	Used	Used	<50%
Macke et al. (16)^*^	sRCT	Prehospital	Germany	2017–2019	152	C-MAC (M or A) vs. DL	≥50%	Experienced	Unknown	Unknown	≥50%
Ilbagi et al. (45)	sRCT	ER	Iran	2016–2018	70	Glidescope (A) vs. DL	<50%	Inexperienced	Used	Used	<50%
Sanguanwit et al. (46)	sRCT	ER	Thailand	2015–2016	158	Glidescope (A) vs. DL	<50%	Inexperienced	Unknown	Unknown	<50%

The meta-analysis and network meta-analysis included all of the listed articles except those indicated with asterisks (^*^). The network meta-analysis compared video laryngoscope blades investigated in the studies.

* These studies were excluded from the network meta-analysis because the video laryngoscopes used could not be classified according to blade type.

** Could not be classified by blade because a fiberoptic video bronchoscope was used.

*** Could not be classified by blade because an endotracheal tube with an integrated camera at the tip was used.

sRCT, single-center randomized controlled trial; mRCT, multicenter randomized controlled trial; ER, emergency room; CPR, cardiopulmonary resuscitation; ICU, intensive care unit; RSI, rapid sequence intubation; A, nonchanneled angular blade; C, channeled blade; M, nonchanneled Macintosh blade; DL, direct laryngoscope.

In the NMA comparing DL with VL, three VL device types, based on their blades, were included: nonchanneled Macintosh blades (C-MAC, McGrath, and UE scope), nonchanneled angular blades (Glidescope), and channeled blades (Airtraq, Airwayscope, and King Vision). The King Vision was only employed as a channeled device in the included studies. However, the VivaSight included in Grensemann's 2018 article was excluded from the NMA based on blade type because of its single-lumen tube with an integrated video camera that could not be categorized according to the criteria used in this review. The articles by Janz et al. (39) and Macke et al. (16) were excluded from the NMA because the VL devices could not be classified by the blade type (Supplementary Table S2).

### 3.2. Risk of bias within studies

Regarding the overall risk of bias in each included study, 11 studies had low risk of bias, seven studies were categorized as *some concerns*, and four studies had high risk. In the detailed assessment of all subcategories, the *randomization processes* of nine studies were rated as *some concerns* or *high risk of bias* because detailed descriptions of the randomization processes were omitted. Additionally, the *deviations from intended interventions* of three studies were classified as *some concerns* because it was unclear whether the exclusions of some members of the study populations occurred before or after randomization. Details of the quality assessments of the included studies are shown in Supplementary Figure S2.

### 3.3. Publication bias across studies

Supplementary Figure S3 presents a funnel plot indicating that the first-pass success comparison between VL and DL was balanced. Egger's regression test revealed no significant bias across studies (*p* = 0.28).

### 3.4. Synthesis of first-attempt success results

Data on first-attempt success rates were provided for all 22 included studies. A pooled analysis revealed no statistically significant difference in the first-attempt success rates between VL and DL (22 studies; OR, 1.16; 95% CI, 0.66–2.03; *n* = 4,244; *p* = 0.62), with significant heterogeneity among studies (*p* < 0.01; I^2^=85%; Supplementary Figure S4).

### 3.5. Sensitivity analysis

An outlier article by Trimmel et al. (34) caused the most heterogeneity and, in comparison with most of the other articles, the article by Trimmel et al. (34) yielded considerably dissimilar outcomes, with lower VL success rates and higher DL success rates. Pooled analysis revealed no significant difference in the first-attempt success rate between VL and DL after eliminating the outlier study from the sensitivity analysis (21 studies; success rate, VL vs. DL, 77.3% vs. 75.3%, respectively; OR, 1.36; 95% CI, 0.84–2.20; *n* = 3,918; *p* = 0.20; low-quality evidence), with significant study heterogeneity (*p* < 0.01; I^2^ = 80%; Supplementary Figure S4).

### 3.6. Subgroup analyses

**Figure 2:**
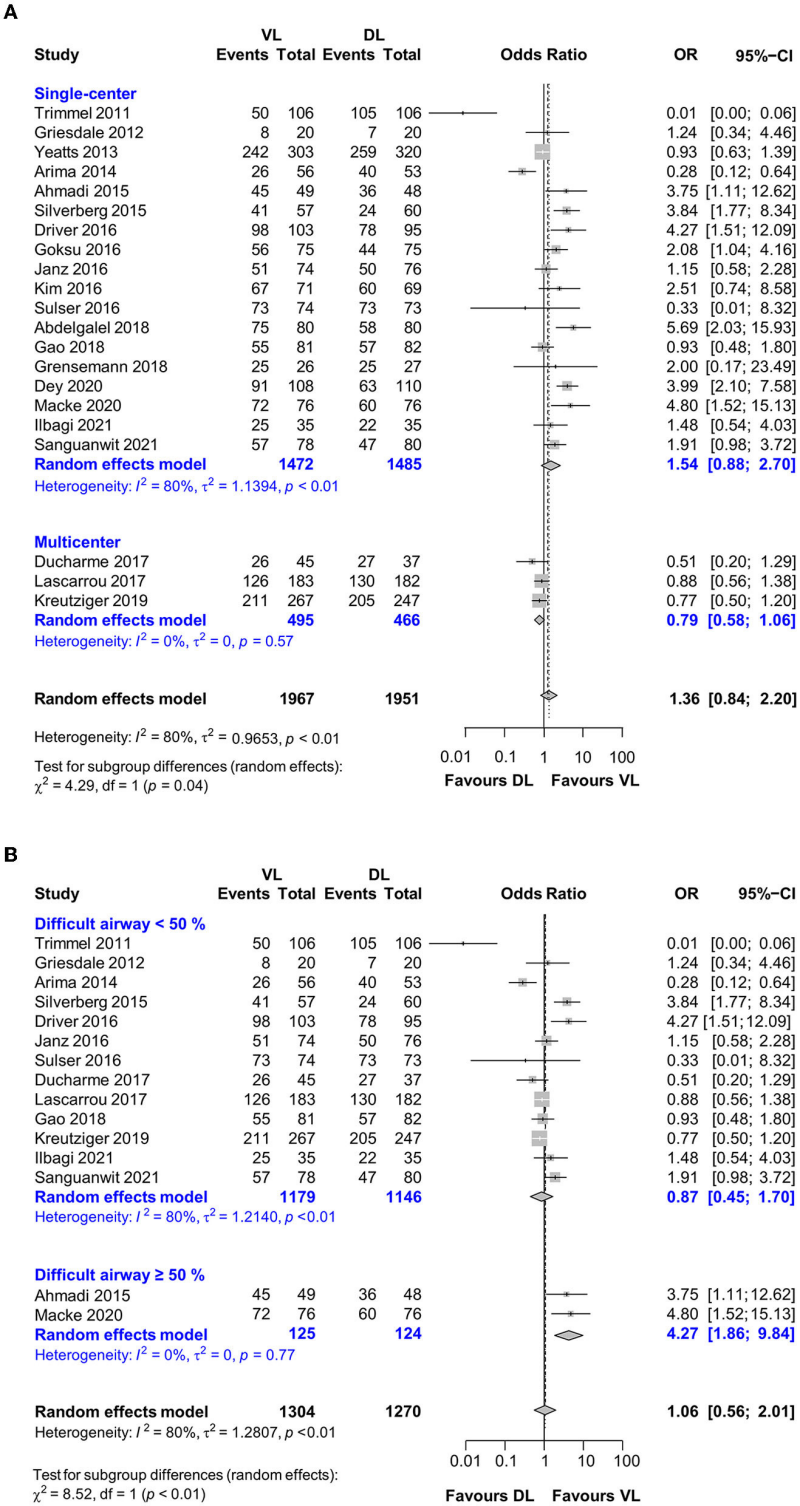
Forest plot depicting the subgroup analysis for **(A)** study design and **(B)** difficult airway.

After sensitivity analysis, 21 studies underwent subgroup analyses to compare first-attempt intubation success between VL and DL. Significant heterogeneity within studies was only apparent in terms of two factors: study design and difficult airway proportion. The heterogeneity-producing effect of study design was compared between single-center studies (18 studies; *n* = 2,957; OR, 1.54; 95% CI, 0.88–2.70; low-quality evidence) and multicenter studies (three studies; *n* = 961; success rate, VL vs. DL, 73.3% vs. 77.7%, respectively; OR, 0.79; 95% CI, 0.58–1.06; high-quality evidence; Figure 2A). This showed that the pooled outcome of multicenter RCTs was more consistent than that of single-center RCTs (I^2^ heterogeneity: single center vs. multicenter, 80% vs. 0%, *p* = 0.04). After excluding six articles that did not report the proportions of difficult airways, the subgroup analysis for difficult airways included 15 studies, with a significant heterogeneity effect in the comparison of difficult airway proportions between <50% and ≥50% (*p* < 0.01). Most studies included <50% difficult airways, and the pooled results showed that VL had the same success rate as DL (13 studies; *n* = 2,325; OR, 0.87; 95% CI, 0.45–1.70; I^2^ = 80%; low-quality evidence). In contrast, pooled results from studies including ≥50% difficult airways showed a higher success rate for VL than DL (two studies; *n* = 249; success rate, 93.6% vs. 77.4%; OR, 4.27; 95% CI, 1.86–9.84; I^2^ = 0%; moderate-quality evidence; Figure 2B).

Other factors suspected of causing heterogeneity were intubators' experience (inexperienced vs. experienced, *p* = 0.48), rapid sequence intubation (yes vs. no, *p* = 0.32), and the proportion of intubation during CPR (<50% vs. ≥50%, *p* = 0.45). VL was associated with a higher success rate than DL, with moderate-quality evidence, when used by inexperienced nonphysicians (11 studies; *n* = 2,050; success rate, 75.8% vs. 70.4%; OR, 1.54; 95% CI, 1.04–2.26; I^2^ = 64%) or in-hospital settings (16 studies; *n* = 2,849; success rate, 80.1% vs. 72.1%; OR, 1.86; 95% CI, 1.32–2.64; I^2^ = 65%).

In the analysis using meta-regression, the recruitment start dates and sample sizes were not significant (21 studies; recruitment period, *p* = 0.11; sample size, *p* = 0.40; Supplementary Tables S4, S5).

### 3.7. NMA by blade type

The effect of VL blade type for the first-attempt intubation success was evaluated using Bayesian NMA (18 studies; *n* = 3,563) for three VL blade types: nonchanneled Macintosh VL vs. nonchanneled angular VL vs. channeled VL; DL as a reference treatment). The best Bayesian NMA model in this comparison was obtained to minimize inconsistency (deviance information criterio*n* = 72.2; I^2^ = 6%).

**Figure 3:**
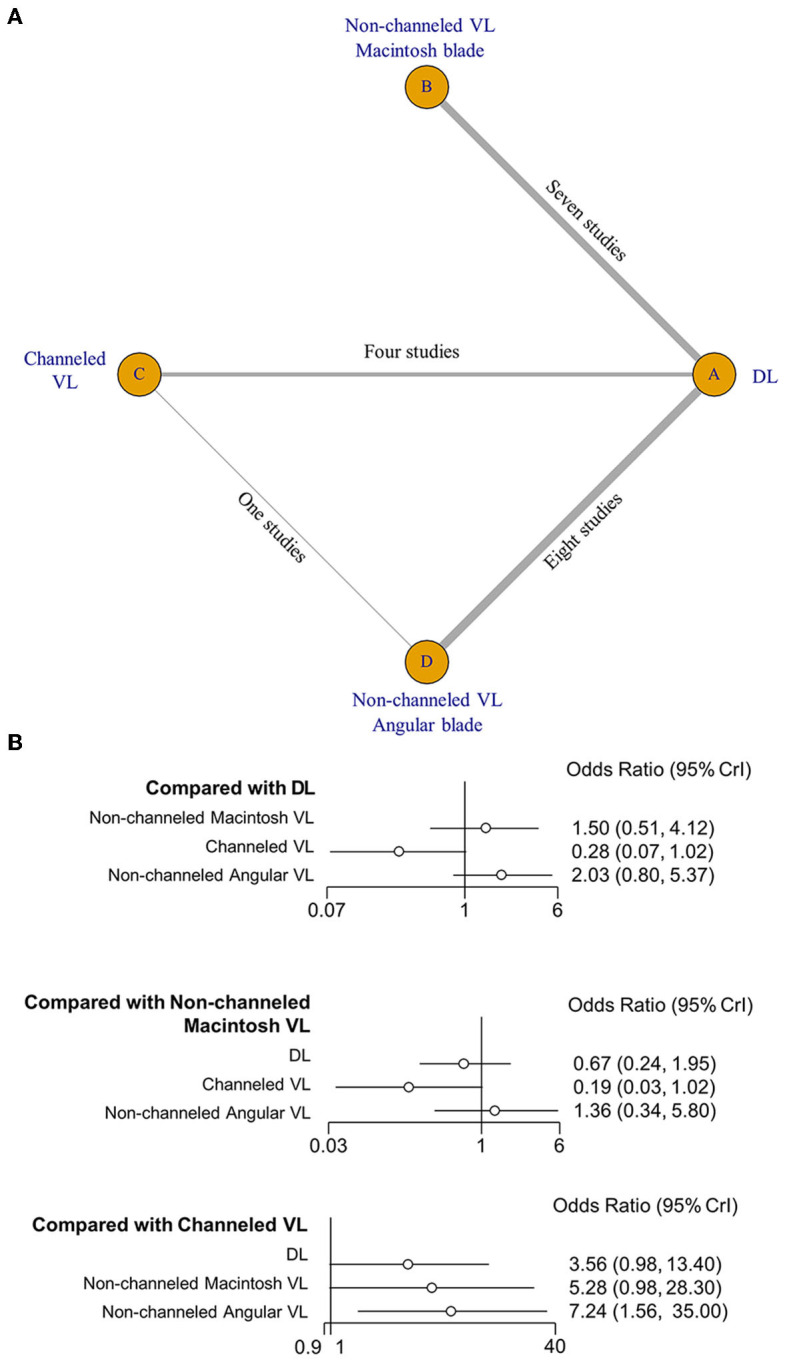
Network map **(A)** and forest plot **(B)** of the network meta-analysis of first-attempt intubation success.

The network graph for first-pass success revealed that three types of VLs and DL could be directly compared. There was only one indirect comparison between channeled VL and nonchanneled angular VL in the VL intercomparison (Figure 3A). Figure 3B shows that the nonchanneled Macintosh and angular blades of nonchanneled VL had an intubation success rate similar to that of DL (nonchanneled Macintosh VL, OR, 1.50; 95% CI, 0.51–4.12; nonchanneled angular VL, OR, 2.03; 95% CI, 0.80–5.37). However, channeled VL had a relatively lower intubation success rate than DL (OR, 0.28; 95% CI, 0.08–1.02). In a VL intercomparison, nonchanneled angular VL had a significantly higher intubation success rate than channeled VL (OR, 7.24; 95% CI, 1.56–35.0).

According to the rank probability test results in Table 2, the nonchanneled angular VL had the best outcome (0.669), the nonchanneled Macintosh VL was ranked second (0.472), and DL third (0.727). The fourth-ranked treatment (0.958) was channeled VL.

**Table 2:** Rank probability of laryngoscope blade efficacy.

**Laryngoscope blade**	**Rank probability**			
	**1st**	**2nd**	**3rd**	**4th**
DL	0.017	0.238	**0.727**	0.018
Nonchanneled Macintosh VL	0.311	**0.472**	0.195	0.022
Nonchanneled Angular VL	**0.669**	0.278	0.050	0.003
Channeled VL	0.003	0.012	0.028	**0.958**

DL, direct laryngoscope; VL, video laryngoscope. The bold values are the highest value among the each column.

## 4. Discussion

This pooled analysis found, with a low certainty of evidence, that VL, compared with DL, does not improve ETI success in critically ill or emergency-care patients. This finding was supported by pooled results from multicenter RCTs with a high certainty of evidence via sensitivity and subgroup analyses. Using a subgroup approach, we found that VL outperformed DL in association with difficult airways, inexperienced practitioners, and in-hospital settings, with a moderate certainty of evidence. The patient's airway status, intubator experience with laryngoscopes, and intubator surroundings were all significantly associated with ETI success in the evaluation of VL efficacy. In the selection of VL for ETI, this study revealed that channeled VLs had a lower efficacy of ETI success compared with nonchanneled VLs and DL.

VL has advantages for use in the intubation of patients with difficult airways; however, in this review, only a few studies had a high proportion (>50%) of difficult airways, and most of the included studies included a relatively low number of difficult airways (Figure 2), which is the main reason for the equivalent results of VL and DL. Moreover, it might be challenging to include mainly patients who have difficult airways with a high risk of ETI failure in each RCT. Thus, the pooled result of this review may have differed if more studies with high proportions of difficult airways were included.

This review did not demonstrate that differences in ETI providers' levels of experience could affect the success of laryngoscopy (test for subgroup difference in Supplementary Figure S5; experienced vs. inexperienced group, *p* = 0.48). VL showed equal ETI performance in the experienced group (success rate: VL, 745/945 (78.8%); DL, 746/923 (80.8%); OR, 1.00; 95% CI, 0.33–3.09; I^2^ = 87%), but outperformed DL in the inexperienced intubator group, with a moderate certainty of evidence [success rate: VL, 775/1,022 (75.8%); DL, 724/1,028 (70.4%); OR, 1.54; 95% CI, 1.04–2.26; I^2^ = 64%]. The similarities in the ETI success rates of DL and VL in the total population and experienced group were possibly attributable to an imbalance in ETI experience between VL and DL providers. This implies a significant gap between DL providers' sufficient ETI experience and VL providers' insufficient experience. Most of the included studies revealed that ETI experience for VL providers was limited to manikin training rather than actual patients (Supplementary Table S2) (34, 42, 44). This disparity in ETI experience may cause the DL-associated ETI success rate to be higher than the VL-associated success rate. Some studies with high heterogeneity in the Baujat plot demonstrated this effect, wherein experienced intubators performed ETIs in the prehospital setting, resulting in an apparently higher rate of ETI success with DL than with VL (Supplementary Figure S4) (15, 34, 37). When interpreting the pooled results of this meta-analysis, consideration of the hidden disparity in ETI experience, rather than an exclusive focus on statistical results, is crucial.

Channeled VL had a lower success rate in an NMA relative to nonchanneled VLs and DL. The obvious structural difference between channeled VL and DL could be the primary reason for the lower success rate of channeled VLs. Nonchanneled VL has structural similarities to DL, as well as the use of a stylet in combination with the endotracheal tube. However, ETI using channeled VL can be unfamiliar because it is an alternative ETI procedure without stylet use. The channeled VL blade is slightly thicker than that used in DL to guide the advancement of the tracheal tube during ETI, and this likely increases the difficulty of ETI in patients with narrow or restricted oral cavities (37, 47). Given the intubators' considerable familiarity with DL, the unfamiliar structure and lack of VL experience could be the primary reasons for the channeled VL's lower ETI performance.

There was no statistical difference in ETI between VL and DL in the total population (OR 1.16, CI 0.66–2.03), and heterogeneity was high (I^2^ = 85%); thus, the certainty of evidence was low. Sensitivity analysis, subgroup analysis, and meta-regression were used to reduce heterogeneity and obtain more consistent results.

In the sensitivity analysis, omitting the 2016 article by Trimmel et al. (15) significantly reduced the heterogeneity of the pooled results (up to I^2^ = 80%, *p* < 0.01). The study by Trimmel et al. (15) contributed the most heterogeneity, according to the Baujat plot, indicating that this study was a significant outlier and demonstrating DL's higher success rate relative to that of VL (Supplementary Figure S4). Thus, the greater experience of practitioners in Trimmel's 2016 study (a mean of 7 years of anesthesiology experience) compared with other included studies, as well as the ground and air ambulance prehospital settings wherein ETI was performed, were the main reasons for its role as a significant outlier.

We anticipated that the intubator surroundings would affect VL efficacy. VL performed similarly to DL in the prehospital setting, but it outperformed DL in the in-hospital setting. Despite the fact that this subgroup categorization was not associated with statistical significance (*p* = 0.10), differences in intubator surroundings could affect ETI performance. Most prehospital studies reported the following unique reasons for ETI failure in VL: impaired sight due to ambient light, fogged camera lenses, and monitor problems. Furthermore, these studies revealed a high rate of arrest, ETI during CPR, oral contamination, and cervical immobilization in trauma patients (13, 34, 40). We predicted that these characteristics of prehospital surroundings would be more detrimental to VL than DL.

Sensitivity analysis, subgroup analysis, and meta-regression did not significantly help reduce heterogeneity, mostly owing to the high rate of single-center RCTs. Differences between recruited hospitals, such as the severity of patients' conditions, hospital size or capacity, and clinicians' skill or experience, may have influenced the results of the pooled analysis. The pooled analysis of multicenter RCTs showed consistent results with a high certainty of evidence (three studies; *n* = 961; OR, 0.79; 95% CI, 0.58–1.06; I^2^ = 0%; Figure 1, Supplementary Table S4). Thus, more multicenter RCTs are needed to obtain reliable results.

This meta-analysis yielded the same conclusion as a previous meta-analysis by Jiang et al.: VL was not related to a higher success rate compared with DL in the total population (1). Jiang et al. also reported that prehospital intubation is worsened by VL use even when it is performed by experienced operators. However, this pooled result differed from our analysis. By adding more recently completed studies, we demonstrated VL to be equally successful as DL in the prehospital ETI or experienced intubator group (1), possibly due to the definition of an experienced intubator.

Regarding ETI experience among intubation providers, Jiang et al. included inconsistent criteria for experienced operators (including certified anesthesiologists, emergency medical technicians with >3 years of clinical experience, personnel who performed >50 ETIs, or according to the judgment of the study investigators), and these were primarily related to DL and did not depend on experience with ETI using VL. In contrast to the study conducted by Jiang et al. (1), experienced intubators in the present analysis were designated as physicians with sufficient experience, whereas the inexperienced group included students, paramedics, nurses, residents, and fellow trainees. Although this definition did not completely standardize the difference in intubator experience between DL and VL providers in each of the included studies, it likely lessened the significant heterogeneity of analysis caused by ambiguous criteria defining experienced intubators.

Vargas et al. recently published a systematic review and meta-analysis demonstrating that VL increased the rate of successful ETI on the first attempt relative to the rate achieved with DL (RR 1.04, 95% CI, 1.01–1.08, I^2^= 79%) (48). Vargas et al. reported that VL outperformed DL in overall results, but their study included a different population than our review because they included surgical patients but excluded those who needed prehospital ETI or ETI during CPR. Therefore, the population differences between these studies may have affected the pooled results (49). During CPR, the camera view of a VL may frequently become obscured due to fogging, secretions, blood, or emesis present in the oropharynx. Consequently, the success rate of ETI using a VL during CPR may be considerably lower in comparison to that achieved using a DL (50). Moreover, in the prehospital setting, where airway management procedures may be conducted outdoors, the presence of sun glare can also impede the successful ETI (51). It is, therefore, plausible that these differences among the studies may have influenced the pooled results.

We believe that this study revealed a scarcity of articles demonstrating the true efficacy of VL because there was a significant gap in ETI experience between VL and DL. The lack of experience for VL is strongly related to the current status of VL, which is only used as a rescue device in difficult airways. This is seriously impeding the accumulation of VL intubating experience (52). This study's VL inexperience is synthesized as a lack of training, familiarity, confidence as a result of occasional use, and knowledge of ETI indication. Because the factors mentioned are significantly associated with insufficient years of ETI experience, years of ETI experience is regarded as the most important factor for ETI success. We also believe that the absence of support from a well-trained technician or nurse outside of the hospital is a major factor impeding overall ETI success. To address this issue, some recent articles suggest that a shift from DL to VL as a legal standard of care is necessary, which can help increase ETI success rates as well as VL's rapid accumulation of experience (50, 51). In this context, future trials by expert clinicians with sufficient real-world experience in both VL and DL, such as non-channeled angular VL showing the best performance in the rank probability test, can thus demonstrate the true efficacy of VL in difficult airways.

Several previous studies have reported that VL improves the first-pass success rates and reduces the risk of ETI failure in patients with difficult airways (50, 51). In the subgroup analysis of the present meta-analysis, which included only two studies, the pooled results of studies with ≥50% difficult airways demonstrated a higher success rate for VL than for DL. In the rank probability of laryngoscope blade efficacy, two VL (nonchanneled angular VL and nonchanneled Macintosh VL) was ranked first and second devices respectively, in contrast, DL was ranked third devices. VL has been shown to be advantageous in managing difficult airways by providing better visualization of the airway structures, which can result in more successful ETI and fewer complications. Although in the ETI trial, VL may have been designated as a backup device, the results of our analysis suggest that VL could be considered as one of the primary tools for ETI in patients with predicted difficulty airway.

There are several limitations to this review and meta-analysis. First, our study aimed to establish a strict standard for ETI experience to minimize heterogeneity; however, this standard did not entirely eliminate the heterogeneity results. The measures of ETI experience, such as manikin training, clinical department experience, and ETI times threshold, varied among the included studies for both DL and VL. Furthermore, in the context of VL experience, a shortage of training time with VL and the use of manikins during training may have contributed to the lower success rate observed with VL. To improve the outcomes and heterogeneity of future RCTs, it is necessary to resolve these potential confounding factors. Second, rather than a fixed model, we used a random-effects model to account for the diverse medical resources or environments; however, a random-effects model cannot completely resolve hidden heterogeneity issues, including information gaps and selection bias. Third, most of the studies included in this meta-analysis did not provide sufficient information regarding the patients' disease, therefore, the assessment of the severity of their condition was not evaluated in this meta-analysis. Although time to ETI was not the primary outcome in this meta-analysis, it may be a crucial factor in patients with poor oxygen reserves or high oxygen demands, such as those with sepsis. In these patient populations, who present with physiological challenges, selecting the laryngoscope device that offers the highest success rate and the shortest time to ETI should be chosen. Finally, the population included in this study exhibits a substantial degree of heterogeneity, characterized by variations in disease severity, difficult airway, ETI location (pre-hospital or in-hospital), ETI during CPR, the presence of additional support during ETI such as a nurse, and the type of laryngoscopes employed. This diversity is reflected in the calculated heterogeneity. the quality of evidence supporting the comparison of multiple devices is considered low. Given these limitations of this study, the findings of the meta-analysis should be interpreted with caution.

## 5. Conclusion

This pooled analysis showed, with a low certainty of evidence, that VL does not improve intubation success compared with DL; however, VL outperformed DL in the contexts of difficult airways, inexperienced ETI providers, and in-hospital settings, with a moderate certainty of evidence. Channeled VL had the least efficacy for intubation success compared with nonchanneled VLs and DL.

## Data availability statement

The raw data supporting the conclusions of this article will be made available by the authors, without undue reservation.

## Author contributions

JK, CA, and WK developed the concept, performed the data analysis, and drafted the manuscript. JK and B-HJ performed data acquisition. CA and WK performed the statistical analysis. All authors approved the final version.

## References

[1] Jiang J Ma D Li B Yue Y Xue F. Video laryngoscopy does not improve the intubation outcomes in emergency and critical patients - a systematic review and meta-analysis of randomized controlled trials. Crit Care. (2017) 21:288. 10.1186/s13054-017-1885-9 29178953 PMC5702235

[2] Sulser S Ubmann D Schlaepfer M Brueesch M Goliasch G Seifert B . C-MAC videolaryngoscope compared with direct laryngoscopy for rapid sequence intubation in an emergency department: A randomised clinical trial. Eur J Anaesthesiol. (2016) 33:943–8. 10.1097/EJA.0000000000000525 27533711

[3] Silverberg MJ Li N Acquah SO Kory PD. Comparison of video laryngoscopy versus direct laryngoscopy during urgent endotracheal intubation: a randomized controlled trial. Crit Care Med. (2015) 43:636–41. 10.1097/CCM.0000000000000751 25479112

[4] Brown CA 3rd Bair AE Pallin DJ Walls RM NEAR III Investigators. Techniques, success, and adverse events of emergency department adult intubations. Ann Emerg Med. (2015) 65:363–370.e1. 10.1016/j.annemergmed.2014.10.036 25533140

[5] Jaber S Amraoui J Lefrant JY Arich C Cohendy R Landreau L . Clinical practice and risk factors for immediate complications of endotracheal intubation in the intensive care unit: a prospective, multiple-center study. Crit Care Med. (2006) 34:2355–61. 10.1097/01.CCM.0000233879.58720.87 16850003

[6] Lakticova V Koenig SJ Narasimhan M Mayo PH. Video laryngoscopy is associated with increased first pass success and decreased rate of esophageal intubations during urgent endotracheal intubation in a medical intensive care unit when compared to direct laryngoscopy. J Intensive Care Med. (2015) 30:44–8. 10.1177/0885066613492641 23771876

[7] Soar J Böttiger BW Carli P Couper K Deakin CD Djärv T . European Resuscitation Council Guidelines 2021: adult advanced life support. Resuscitation. (2021) 161:115–51. 10.1016/j.resuscitation.2021.02.010 33773825

[8] Wang HE Kupas DF Hostler D Cooney R Yealy DM Lave JR. Procedural experience with out-of-hospital endotracheal intubation. Crit Care Med. (2005) 33:1718–21. 10.1097/01.CCM.0000171208.07895.2A 16096447

[9] Rajajee V Riggs B Seder DB. Emergency neurological life support: airway, ventilation, and sedation. Neurocrit Care. (2017) 27:4–28. 10.1007/s12028-017-0451-2 28913751

[10] Lascarrou JB Boisrame-Helms J Bailly A Le Thuaut A Kamel T Mercier E . Clinical Research in Intensive Care and Sepsis (CRICS) Group. Video laryngoscopy vs direct laryngoscopy on successful first-pass orotracheal intubation among ICU patients: A randomized clinical trial. JAMA. (2017) 317:483–93. 10.1001/jama.2016.20603 28118659

[11] Mosier JM Sakles JC Stolz U Hypes CD Chopra H Malo J . Neuromuscular blockade improves first-attempt success for intubation in the intensive care unit. A propensity matched analysis. Ann Am Thorac Soc. (2015) 12:734–41. 10.1513/AnnalsATS.201411-517OC 25719512 PMC5466156

[12] Jaber S Jung B Corne P Sebbane M Muller L Chanques G . An intervention to decrease complications related to endotracheal intubation in the intensive care unit: a prospective, multiple-center study. Intensive Care Med. (2010) 36:248–55. 10.1007/s00134-009-1717-8 19921148

[13] Kreutziger J Hornung S Harrer C Urschl W Doppler R Voelckel WG . Comparing the McGrath Mac video laryngoscope and direct laryngoscopy for prehospital emergency intubation in air rescue patients: a multicenter, randomized, controlled trial. Crit Care Med. (2019) 47:1362–70. 10.1097/CCM.0000000000003918 31389835 PMC6791500

[14] Cavus E Janssen S Reifferscheid F Caliebe A Callies A von der Heyden M . Videolaryngoscopy for physician-based, prehospital emergency intubation: A prospective, randomized, multicenter comparison of different blade types using AP Advance, C-MAC System, and KingVision. Anesth Analg. (2018) 126:1565–74. 10.1213/ANE.0000000000002735 29239965

[15] Trimmel H Kreutziger J Fitzka R Szüts S Derdak C Koch E . Use of the GlideScope Ranger video laryngoscope for emergency intubation in the prehospital setting: a randomized control trial. Crit Care Med. (2016) 44:e470–6. 10.1097/CCM.0000000000001669 27002277

[16] Macke C Gralla F Winkelmann M Clausen JD Haertle M Krettek C . Increased first pass success with C-MAC videolaryngoscopy in prehospital endotracheal intubation-a randomized controlled trial. J Clin Med. (2020) 9:2719. 10.3390/jcm9092719 32842705 PMC7564813

[17] Kim JW Park SO Lee KR Hong DY Baek KJ Lee YH . Video laryngoscopy vs direct laryngoscopy: which should be chosen for endotracheal intubation during cardiopulmonary resuscitation? A prospective randomized controlled study of experienced intubators. Resuscitation. (2016) 105:196–202. 10.1016/j.resuscitation.2016.04.003 27095126

[18] Driver BE Prekker ME Moore JC Schick AL Reardon RF Miner JR. Direct versus video laryngoscopy using the C-MAC for tracheal intubation in the emergency department, a randomized controlled trial. Acad Emerg Med. (2016) 23:433–9. 10.1111/acem.12933 26850232

[19] Teoh WH Saxena S Shah MK Sia AT. Comparison of three videolaryngoscopes: pentax Airway Scope, C-MAC, Glidescope vs. the Macintosh laryngoscope for tracheal intubation. Anaesthesia. (2010) 65:1126–32. 10.1111/j.1365-2044.2010.06513.x 20883502

[20] Kleine-Brueggeney M Greif R Schoettker P Savoldelli GL Nabecker S Theiler LG. Evaluation of six videolaryngoscopes in 720 patients with a simulated difficult airway: a multicentre randomized controlled trial. Br J Anaesth. (2016) 116:670–9. 10.1093/bja/aew058 27106971

[21] Griesdale DE Liu D McKinney J Choi PT. Glidescope® video-laryngoscopy versus direct laryngoscopy for endotracheal intubation: a systematic review and meta-analysis. Can J Anaesth. (2012) 59:41–52. 10.1007/s12630-011-9620-5 22042705 PMC3246588

[22] Noppens RR Geimer S Eisel N David M Piepho T. Endotracheal intubation using the C-MAC® video laryngoscope or the Macintosh laryngoscope: a prospective, comparative study in the ICU. Crit Care. (2012) 16:R103. 10.1186/cc11384 22695007 PMC3580658

[23] Goksu E Kilic T Yildiz G Unal A Kartal M. Comparison of the C-MAC video laryngoscope to the Macintosh laryngoscope for intubation of blunt trauma patients in the ED. Turk J Emerg Med. (2016) 16:53–6. 10.1016/j.tjem.2016.02.001 27896321 PMC5121268

[24] De Jong A Molinari N Conseil M Coisel Y Pouzeratte Y Belafia F . Video laryngoscopy versus direct laryngoscopy for orotracheal intubation in the intensive care unit: a systematic review and meta-analysis. Intensive Care Med. (2014) 40:629–39. 10.1007/s00134-014-3236-5 24556912

[25] Lee J Cho Y Kim W Choi KS Jang BH Shin H . Comparisons of videolaryngoscopes for intubation undergoing general anesthesia: systematic review and network meta-analysis of randomized controlled trials. J Pers Med. (2022) 12:363. 10.3390/jpm12030363 35330362 PMC8954588

[26] Hutton B Salanti G Caldwell DM Chaimani A Schmid CH Cameron C . The PRISMA extension statement for reporting of systematic reviews incorporating network meta-analyses of health care interventions: checklist and explanations. Ann Intern Med. (2015) 162:777–84. 10.7326/M14-2385 26030634

[27] Sterne JA Savović J Page MJ Elbers RG Blencowe NS Boutron I . RoB 2: a revised tool for assessing risk of bias in randomised trials. BMJ. (2019) 366:l4898. 10.1136/bmj.l4898 31462531

[28] Egger M Davey Smith G Schneider M Minder C. Bias in meta-analysis detected by a simple, graphical test. BMJ. (1997) 315:629–34. 10.1136/bmj.315.7109.629 9310563 PMC2127453

[29] Guyatt G Oxman AD Akl EA Kunz R Vist G Brozek J . GRADE guidelines: 1. Introduction-GRADE evidence profiles and summary of findings tables. J Clin Epidemiol. (2011) 64:383–94. 10.1016/j.jclinepi.2010.04.026 21195583

[30] Baujat B Mahé C Pignon JP Hill C A. graphical method for exploring heterogeneity in meta-analyses: application to a meta-analysis of 65 trials. Stat Med. (2002) 21:2641–52. 10.1002/sim.1221 12228882

[31] van Valkenhoef G Lu G de Brock B Hillege H Ades AE Welton NJ. Automating network meta-analysis. Res Synth Methods. (2012) 3:285–99. 10.1002/jrsm.1054 26053422

[32] Dias S Welton NJ Sutton AJ Caldwell DM Lu G Ades AE. NICE DSU Technical Support Document 4: Inconsistency in Networks of Evidence Based on Randomised Controlled Trials. London: National Institute for Health and Care Excellence (NICE). (2014). 27466656

[33] Salanti G Ades AE Ioannidis JP. Graphical methods and numerical summaries for presenting results from multiple-treatment meta-analysis: an overview and tutorial. J Clin Epidemiol. (2011) 64:163–71. 10.1016/j.jclinepi.2010.03.016 20688472

[34] Trimmel H Kreutziger J Fertsak G Fitzka R Dittrich M Voelckel WG. Use of the Airtraq laryngoscope for emergency intubation in the prehospital setting: a randomized control trial. Crit Care Med. (2011) 39:489–93. 10.1097/CCM.0b013e318206b69b 21169822

[35] Griesdale DE Chau A Isac G Ayas N Foster D Irwin C . Canadian Critical Care Trials Group. Video-laryngoscopy versus direct laryngoscopy in critically ill patients: a pilot randomized trial. Can J Anaesth. (2012) 59:1032–9. 10.1007/s12630-012-9775-8 22932944

[36] Yeatts DJ Dutton RP Hu PF Chang YW Brown CH Chen H . Effect of video laryngoscopy on trauma patient survival: a randomized controlled trial. J Trauma Acute Care Surg. (2013) 75:212–9. 10.1097/TA.0b013e318293103d 23823612

[37] Arima T Nagata O Miura T Ikeda K Mizushima T Takahashi A . Comparative analysis of airway scope and Macintosh laryngoscope for intubation primarily for cardiac arrest in prehospital setting. Am J Emerg Med. (2014) 32:40–3. 10.1016/j.ajem.2013.09.026 24176585

[38] Ahmadi K Ebrahimi M Hashemian AM Sarshar S Rahimi-Movaghar V. GlideScope video laryngoscope for difficult intubation in emergency patients: a quasi-randomized controlled trial. Acta Med Iran. (2015) 53:738–42. 26749229

[39] Janz DR Semler MW Lentz RJ Matthews DT Assad TR Norman BC . Facilitating EndotracheaL intubation by Laryngoscopy technique and apneic Oxygenation Within the ICU Investigators and the Pragmatic Critical Care Research Group. Randomized trial of video laryngoscopy for endotracheal intubation of critically ill adults. Crit Care Med. (2016) 44:1980–7. 10.1097/CCM.0000000000001841 27355526 PMC5203695

[40] Ducharme S Kramer B Gelbart D Colleran C Risavi B Carlson JN . pilot, prospective, randomized trial of video versus direct laryngoscopy for paramedic endotracheal intubation. Resuscitation. (2017) 114:121–6. 10.1016/j.resuscitation.2017.03.022 28336412

[41] Abdelgalel EF Mowafy SM. Comparison between Glidescope, Airtraq and Macintosh laryngoscopy for emergency endotracheal intubation in intensive care unit: randomized controlled trial. Egypt J Anaesth. (2018) 34:123–8. 10.1016/j.egja.2018.06.002

[42] Gao YX Song YB Gu ZJ Zhang JS Chen XF Sun H . Video versus direct laryngoscopy on successful first-pass endotracheal intubation in ICU patients. World J Emerg Med. (2018) 9:99–104. 10.5847/wjem.j.1920-8642.2018.02.003 29576821 PMC5847508

[43] Grensemann J Eichler L Wang N Jarczak D Simon M Kluge S. Endotracheal tube-mounted camera-assisted intubation versus conventional intubation in intensive care: a prospective, randomised trial (VivaITN). Crit Care. (2018) 22:235. 10.1186/s13054-018-2152-4 30241488 PMC6151025

[44] Dey S Pradhan D Saikia P Bhattacharyya P Khandelwal H Adarsha KN. Intubation in the Intensive Care Unit: C-MAC video laryngoscope versus Macintosh laryngoscope. Med Intensiva (Engl Ed). (2020) 44:135–41. 10.1016/j.medin.2019.10.004 31780257

[45] Ilbagi M Nasr-Esfahani M. The efficacy of using video laryngoscopy on tracheal intubation by novice physicians. Iran J Otorhinolaryngol. (2021) 33:37–44. 10.22038/ijorl.2020.43797.2447 33654689 PMC7897431

[46] Sanguanwit P Yuksen C Laowattana N. Direct versus video laryngoscopy in emergency intubation: A randomized control trial study. Bull Emerg Trauma. (2021) 9:118–24. 10.30476/BEAT.2021.89922.1240 34307701 PMC8286653

[47] Otsuka Y Hirabayashi Y Taga N Takeuchi M Seo N. Use of the airway scope for difficult airway. Masui. (2008) 57:725–7. 18546901

[48] Vargas M Servillo G Buonanno P Iacovazzo C Marra A Putensen-Himmer G . Video vs.. direct laryngoscopy for adult surgical and intensive care unit patients requiring tracheal intubation: a systematic review and meta-analysis of randomized controlled trials. Eur Rev Med Pharmacol Sci. (2021) 25:7734–49. 10.26355/eurrev_202112_27620 34982435

[49] El-Radaideh K Dheeb E Shbool H Garaibeh S Bataineh A Khraise W . Evaluation of different airway tests to determine difficult intubation in apparently normal adult patients: undergoing surgical procedures. Patient Saf Surg. (2020) 14:43. 10.1186/s13037-020-00263-5 33292451 PMC7681946

[50] Asai T Jagannathan N. Videolaryngoscopy is extremely valuable, but should it be the standard for tracheal intubation? Anesth Analg. (2023) 136:679–82. 10.1213/ANE.0000000000006313 36928153

[51] Aziz MF Berkow L. Pro-con debate: videolaryngoscopy should be standard of care for tracheal intubation. Anesth Analg. (2023) 136:683–8. 10.1213/ANE.0000000000006252 36928154

[52] Van Zundert A Pieters B. Videolaryngoscopy: the new standard for intubation. Ten years' experience. Minerva Anestesiol. (2015) 81:1159–62. 26339751

