# Supplementary material for: Comparison of video laryngoscopy with direct laryngoscopy for intubation success in critically ill patients: a systematic review and Bayesian network meta-analysis
Source: Front Med (Lausanne). 2023 Jun 9;10:1193514. doi: 10.3389/fmed.2023.1193514 (PMC10289197; doi:10.3389/fmed.2023.1193514)
Supplement: Supplementary file 1 [file Data_Sheet_1.docx]

**Supplementary file**

**Table S1.** Search Strategy

**Table S2.** Detailed characteristics of included studies

**Table S3.** List of excluded references after full-text review (147 articles)

**Table S4.** GRADE profile for assessing quality of evidence for the included studies for outcomes

**Figure S1.** Type of laryngoscopes

**Figure S2.** Graph of quality assessment of included studies

**Figure S3.** Funnel plot assessing publication bias for outcome

**Figure S4.** Forest plot and heterogeneity plot for first attempt success between direct and video laryngoscope

**Figure S5.** Forest plot in subgroup analysis for all factors except study design and difficult airway

**Table S1.** Search Strategy

Search date: 30 October, 2022

| Database | Search term |
| --- | --- |
| Medline  (via OVID interface) | 1. exp Intubation/ OR intubation.ti,ab,kw. OR intubate.ti,ab,kw. OR exp Laryngoscopes/ OR exp Laryngoscopy/ OR laryngoscop*.ti,ab,kw.  2. exp Emergencies/ OR exp Critical Care/ OR exp Intensive Care Units/ OR exp Critical illness/ OR emergen*.ti,ab,kw.  3. (exp Randomized Controlled Trial/ OR randomized controlled trial.pt. OR randomised controlled trial.pt. OR randomized.ab. OR randomised.ab.) not (animals not (humans and animals)).sh.  4. #1 AND #2 AND #3 |
| EMBASE | 1. 'intubation' OR intubation:ti,ab,kw OR intubate:ti,ab,kw OR 'laryngoscopy' OR 'laryngoscope' OR laryngoscop*:ti,ab,kw  2. 'emergency' OR 'emergency ward' OR emergen*:ti,ab,kw OR 'intensive care' OR 'critical illness'  3. ('randomized controlled trial' OR randomized:pt OR randomised:pt OR (controlled:pt AND trial:pt)) NOT (animals NOT (humans AND animals))  4. #1 AND #2 AND #3 |
| Cochrane library | 1. [MeSH]Intubation OR intubation OR intubate OR [MeSH]Laryngoscopes OR [MeSH]Laryngoscopy OR  2. [MeSH]Emergencies OR [MeSH]Critical Care OR [MeSH]Intensive Care Units OR [MeSH]Critical illness  3. #1 AND #2 |
| Total | 6,185 articles |

**Table S2.** Detailed characteristics of included studies

| Study | Setting | Blades of laryngoscopes | Difficult airway | Intubator’s experience | Sedatives in RSI | Muscle relaxants in RSI |
| --- | --- | --- | --- | --- | --- | --- |
| Trimmel 2011 | Preshospital:  Air and ground ambulance | Airtraq vs. DL:  Airtraq (channeled); DL (unknown) | < 50 %  Inclusion criteria:  Impaired sight due to vomitus, blood, or food bolus; impaired mouth opening | Experienced:  Anesthesiologists and EMS physicians with >3 years of DL experience, ETI > 80 per year, and VL manikin training. | Etomidate  Ketamine  Fentanyl  Midazolam | Succinylcholine |
| Griesdale 2012 | ICU | Glidescope vs. DL:  Glidescope (non-channeled angular 4; DL (Macintosh 3 and 4) | < 50 %  Inclusion criteria:  Airway obstruction; secretion; Mallampati class 3 or 4 | Inexperienced:  Medical students or non-anesthesiology residents with ETI < 5 in the previous 6 months. | Ketamine  Propofol  Fentanyl  Midazolam | Succinylcholine Rocuronium |
| Yeatts 2013 | ER | Glidescope vs. DL:  Glidescope (non-channeled angular; DL (unknown) | Unknown | Inexperienced:  EM or anesthesiology residents with a minimum of 1 year of previous intubations, or a nurse anesthetist, and the attending anesthesiologist (3 % ETI) | Thiopental | Succinylcholine |
| Arima 2014 | Preshospital:  Ground ambulance | Airwayscope vs. DL:  Airwayscope (channeled); DL (unknown) | < 50 %  Inclusion criteria:  Cervical spine immobilization; oral contamination | Experienced:  Anesthesiology (50 % ETI) or other physicians >3 years of ETI experience with the use of both VL and DL in daily practice | Not used | Not used |
| Ahmadi 2015 | ER | Glidescope vs. DL:  Glidescope (non-channeled angular 4); DL (Macintosh 3 and 4) | ≥ 50 %  Inclusion criteria:  Reduced neck extension either pathological or due to immobilization (< 80° from neck flexion); decreased inter-incisor distance (< 3 fingers); short thyromental distance (< 6 cm); Mallampati score 3 or 4; airway obstruction | Inexperienced:  EM residents who lack detailed experience information. | Unknown | Unknown |
| Silverberg 2015 | ICU | Glidescope vs. DL  Glidescope (non-channeled angular 3 and 4); DL (Macintosh 3 and 4, and Miller 4) | < 50 %  Exclusion criteria:  A known history of difficult intubation; presence of limited mouth opening; oropharyngeal masses; swollen tongue | Inexperienced:  Fellow CCM trainees who lack detailed experience information. | Etomidate  (not routinely) | Used but unknown kinds |
| Driver 2016 | ER | C-MAC vs. DL:  C-MAC (non-channeled Macintosh 3 and 4); DL (Macintosh 3 and 4) | < 50 %  The inclusion criteria are not explained in detail. | Inexperienced:  EM residents who lack detailed experience information. | Etomidate  Ketamine | Succinylcholine Rocuronium |
| Goksu 2016 | ER | C-MAC vs. DL:  C-MAC (non-channeled Macintosh 3 and 4); DL (Macintosh 3 and 4) | Unknown | Inexperienced:  EM residents (92 % ETI) and attending physicians (8 % ETI) | Used but unknown kinds | Unknown |
| Janz 2016* | ICU | McGrath, Glidescope and Olympus bronchoscope vs. DL:  McGrath (non-channeled Macintosh); Glidescope (non-channeled angular); Olympus bronchoscope**; DL (Macintosh and Miller) | < 50 %  Inclusion criteria:  Impaired neck mobility; impaired mouth opening; head or neck radiation; airway mass or infection | Inexperienced:  Fellow CCM trainees  Median ETI attempts: VL (68) vs. DL (56)  Median months of fellowship training: VL (23) vs. DL (20) | Etomidate  Ketamine  Propofol  Midazolam | Succinylcholine Rocuronium  Vecuronium |
| Kim 2016 | ER | Glidescope vs. DL:  Glidescope (non-channeled angular); DL (unknown) | Unknown | Experienced:  EM physician with ETI > 50. | Not used | Not used |
| Sulser 2016 | ER | C-MAC vs. DL:  C-MAC (non-channeled Macintosh); DL (Macintosh) | < 50 %  Exclusion criteria:  Major maxillofacial trauma; immobilized cervical spine; indicated awake fiberoptic guided intubation | Experienced:  Anesthesiologists who lack detailed experience information. | Fentanyl  Propofol  Thiopental | Succinylcholine Rocuronium |
| Trimmel 2016 | Preshospital:  Air and ground ambulance | Glidescope vs. DL:  Glidescope (non-channeled angular); DL (unknown) | < 50 %  Inclusion criteria:  Impaired mouth opening; impaired sight due to blood or fluids | Experienced:  EMS physicians with an average of 7 years of anesthesiology experience and VL manikin training. | Etomidate  Ketamine  Fentanyl  Midazolam | Succinylcholine |
| Ducharme 2017 | Preshospital:  Ground ambulance | KingVision vs. DL:  KingVision (channeled); DL (Macintosh and Miller) | < 50 %  Inclusion criteria:  Blood; emesis; obstruction; impaired mouth opening; obesity | Inexperienced:  Paramedics with manikin training experience for both DL and VL | Not used | Not used |
| Lascarrou 2017 | ICU | McGrath vs. Glidescope vs. DL:  McGrath (non-channeled Macintosh); DL (Macintosh) | < 50 %  Inclusion criteria:  Mallampati score 3 or 4; short thyromental distance; impaired mouth opening; obesity | Inexperienced:  Mostly residents (84 % ETI), with a few physicians (16 % ETI). | Etomidate Ketamine Propofol Midazolam | Succinylcholine Rocuronium |
| Abdelgalel 2018 | ICU | Airtraq vs. Glidescope vs. DL:  Airtraq (channeled) vs. Glidescope (non-channeled angular); DL (Macintosh) | Unknown | Experienced:  ICU physicians with > 3 years of anesthesia experience and ETI > 30 for both DL and VL. | Ketamine Propofol  Fentanyl | Rocuronium |
| Gao 2018 | ICU | UEScope vs. DL:  UEScope (non-channeled Macintosh); DL (Macintosh) | < 50 %  Inclusion criteria:  Bleeding; emesis; obstruction; restricted mouth opening; obesity | Experienced:  ICU physicians with > 1 year of experience and manikin training for both DL and VL. | Etomidate  Fentanyl Midazolam | Not used |
| Grensemann 2018* | ICU | VivaSight*** vs. DL (unknown) | Unknown  Inclusion criteria:  Mallampati score of 3 or 4; average thyromental distance; maximum mouth opening average | Experienced:  Attending physicians and fellows with a median of 14 years of experience. | Propofol Sufentanil | Rocuronium |
| Kreutziger 2019 | Preshospital:  Air ambulance | McGrath vs. DL:  McGrath (non-channeled Macintosh); DL (Macintosh) | < 50 %  Inclusion criteria:  Impaired mouth opening; impaired sight due to blood or regurgitation; spine immobilization | Experienced:  EMS physicians who lack detailed experience information. | Fentanyl Esketamine Etomidate Profopol Midazolam | Succinylcholine Rocuronium |
| Dey 2020 | ICU | C-MAC vs. DL:  C-MAC (non-channeled Macintosh 3 and 4); DL (Macintosh 3 and 4) | Unknown | Experienced:  Anesthesiologists with > 3 years of anesthesia experience and ETI > 50 in VL manikin training. | Fentanyl  Propofol Thiopentone | Succinylcholine Rocuronium |
| Macke 2020* | Prehospital:  Air ambulance | C-MAC vs. DL:  C-MAC (non-channeled Macintosh 2-4 or angular D-blade); DL (unknown) | ≥ 50 %  Inclusion criteria:  Facial trauma; impaired mouth opening; impaired sight due to blood or regurgitation; spine immobilization; no neck | Experienced:  EMS physicians with ETI > 100. | Unknown | Unknown |
| Ilbagi 2021 | ER | Glidescope vs. DL:  Glidescope (non-channeled angular 3); DL (Macintosh 3) | < 50 %  Inclusion criteria:  Impaired sight due to blood or regurgitation | Inexperienced:  EM residents who lack detailed experience information. | Fentanyl Etomidate | Succinylcholine |
| Sanguanwit 2021 | ER | Glidescope vs. DL:  Glidescope (non-channeled angular); DL (Macintosh) | < 50 %  Inclusion criteria:  Mallampati score of 3 or 4; abnormal external appearance; Evaluate 3-3-2 abnormal; obstruction; impaired neck mobility | Inexperienced:  Students, residents (75 %), and EM physician (25 %) who lack detailed experience information. | Unknown | Unknown |

*These studies were excluded from network meta-analysis because video laryngoscopes were unable to be classified by each blade.

**Cannot be classified by blade because it was an fiberoptic video bronchoscope

***Cannot be classified by blade because it was an endotracheal tube with an integrated camera at the tip

Abbreviations: EM, emergency medicine; EMS, emergency medical system; DL, direct laryngoscopy; ETI, endotracheal intubation; CCM, critical care medicine; RSI, rapid sequence intubation.

**Table S3.** List of excluded references after full-text review (147 articles)

| Number | Title | First author | Journal (Year) | Main reason for exclusion |
| --- | --- | --- | --- | --- |
| 1 | Effect of the use of an endotracheal tube and stylet versus an endotracheal tube alone on first-attempt intubation success: a multicentre, randomised clinical trial in 999 patients | Jaber et al. | Intensive Care Medicine (2021) | Control group was not eligible for comparison with intervention groups. |
| 2 | First Pass Success Without Adverse Events Is Reduced Equally with Anatomically Difficult Airways and Physiologically Difficult Airways | Pacheco et al. | The Western Journal of Emergency Medicine (2021) | This was not a suitable study design. |
| 3 | Randomized trial of the i-gel supraglottic airway device versus tracheal intubation during out of hospital cardiac arrest (AIRWAYS-2): Patient outcomes at three and six months | Benger et al. | Resuscitation (2020) | Control group was not eligible for comparison with intervention groups. |
| 4 | Head Rotation Reduces Oropharyngeal Leak Pressure of the i-gel and LMA R Supreme TM in Paralyzed, Anesthetized Patients: A Randomized Trial | Chaki et al. | Anesthesia & Analgesia (2021) | Populations did not fulfill. |
| 5 | Prospective, Randomized Comparison of the i-gel and the Self-Pressurized air-Q Intubating Laryngeal Airway in Elderly Anesthetized Patients | Lee et al. | Anesthesia & Analgesia (2020) | Populations did not fulfill. |
| 6 | Videolaryngoscopy in critically ill patients | Jaber et al. | Critical Care (2019) | This was not a suitable study design. |
| 7 | Compared Efficacy of Four Preoxygenation Methods for Intubation in the ICU: Retrospective Analysis of McGrath Mac Videolaryngoscope Versus Macintosh Laryngoscope (MACMAN) Trial Data | Bailly et al. | Critical Care Medicine (2019) | Outcomes did not fulfill. |
| 8 | Effect of a Strategy of a Supraglottic Airway Device vs Tracheal Intubation During Out-of-Hospital Cardiac Arrest on Functional Outcome: The AIRWAYS-2 Randomized Clinical Trial | Banger et al. | JAMA (2018) | Control group was not eligible for comparison with intervention groups. |
| 9 | Effect of a Strategy of Initial Laryngeal Tube Insertion vs Endotracheal Intubation on 72-Hour Survival in Adults With Out-of-Hospital Cardiac Arrest: A Randomized Clinical Trial | Wang et al. | JAMA (2018) | Control group was not eligible for comparison with intervention groups. |
| 10 | A Randomized Comparison of In-hospital Rescuer Positions for Endotracheal Intubation in a Difficult Airway | Le Parc et al. | The Western Journal of Emergency Medicine (2018) | This was not a suitable study design. |
| 11 | Effect of Use of a Bougie vs Endotracheal Tube and Stylet on First-Attempt Intubation Success Among Patients With Difficult Airways Undergoing Emergency Intubation: A Randomized Clinical Trial | Driver et al. | JAMA (2018) | Control group was not eligible for comparison with intervention groups. |
| 12 | Comparison of three different insertion techniques with LMA-Unique TM in adults: results of a randomized trial | Eglen et al. | Revista Brasileira de Anestesiologia (2017) | Control group was not eligible for comparison with intervention groups. |
| 13 | Comparative study of fiberoptic guided versus intubating laryngeal mask airway assisted awake orotracheal intubation in patients with unstable cervical spine | Jadhav et al. | Minerva Anestesiologica (2017) | Populations did not fulfill. |
| 14 | Use of the GlideScope ranger video laryngoscope for emergency intubation in the prehospital setting: a randomized control trial | Trimmel et al. | Critical Care Medicine (2016) | Populations did not fulfill. |
| 15 | Video Laryngoscopy Improves Odds of First-Attempt Success at Intubation in the Intensive Care Unit. A Propensity-matched Analysis | Hypes et al. | Annals of the American Thoracic Society (2016) | This was not a suitable study design. |
| 16 | Comparison of the Airtraq laryngoscope versus the conventional Macintosh laryngoscope while wearing CBRN-PPE | Claret et al. | European Journal of Emergency Medicine (2016) | Populations did not fulfill. |
| 17 | Face-to-face tracheal intubation in adult patients: a comparison of the Airtraq TM, Glidescope TM and Fastrach TM devices | Arslan et al. | Journal of Anesthesia (2015) | Populations did not fulfill. |
| 18 | Higher insertion success with the i-gel supraglottic airway in out-of-hospital cardiac arrest: a randomised controlled trial | Middleton et al. | Resuscitation (2014) | Populations did not fulfill. |
| 19 | Videolaryngoscopy with glidescope reduces cervical spine movement in patients with unsecured cervical spine | Kill et al. | The Journal of Emergency Medicine | Populations did not fulfill. |
| 20 | Comparative study between I-gel, a new supraglottic airway device, and classical laryngeal mask airway in anesthetized spontaneously ventilated patients | Helmy et al. | Saudi Journal of Anaesthesia (2010) | Populations did not fulfill. |
| 21 | Blind tracheal intubation through two supraglottic devices: i-gel versus Fastrach intubating laryngeal mask airway (ILMA) | Sastre et al. | Revista Espanola de Anestesiologia y Reanimacion (2012) | Populations did not fulfill. |
| 22 | LMA Supreme TM vs i-gel TM--a comparison of insertion success in novices | Ragazzi et al. | Anaesthesia (2012) | Populations did not fulfill. |
| 23 | Use of the TrachView videoscope as an adjunct to direct laryngoscopy for teaching orotracheal intubation | Roppolo et al. | European Journal of Emergency Medicine (2012) | This was not a suitable study design. |
| 24 | Out-of-hospital tracheal intubation with single-use versus reusable metal laryngoscope blades: a multicenter randomized controlled trial | Jabre et al. | Annals of Emergency Medicine (2011) | Control group was not eligible for comparison with intervention groups. |
| 25 | A comparison between the Airtraq and Macintosh laryngoscopes for routine airway management by experienced anesthesiologists: a randomized clinical trial | Chalkeidis et al. | Acta Anaesthesiol Taiwan (2010) | Populations did not fulfill. |
| 26 | Comparison of single-use and reusable metal laryngoscope blades for orotracheal intubation during rapid sequence induction of anesthesia: a multicenter cluster randomized study | Amour et al. | Anesthesiology (2010) | Control group was not eligible for comparison with intervention groups. |
| 27 | The GlideScope Ranger video laryngoscope can be useful in airway management of entrapped patients | Nakstad et al. | Acta Anaesthesiologica Scandinavica (2009) | This was not a suitable study design. |
| 28 | Randomized comparison of the classic Laryngeal Mask Airway with the Airway Management Device during anaesthesia | Cook et al. | British Journal of Anaesthesia (2003) | Control group was not eligible for comparison with intervention groups. |
| 29 | Comparison of the lightwand technique with direct laryngoscopy for awake endotracheal intubation in emergency cases | Nishikawa et al. | Journal of Clinical Anesthesia (2001) | Populations did not fulfill. |
| 30 | The intubating laryngeal mask airway compared with direct laryngoscopy | Avidan et al. | British Journal of Anaesthesia (1999) | Control group was not eligible for comparison with intervention groups. |
| 31 | Comparison of the Bullard and Macintosh laryngoscopes for endotracheal intubation of patients with a potential cervical spine injury | Watts et al. | Anesthesiology (1997) | Populations did not fulfill. |
| 32 | Comparison of Macintosh laryngoscope, non- channelled (C-MAC video-laryngoscope) and channelled laryngoscope (Airtraq) for intubation in lateral position – A prospective randomized controlled study | Sultana et al. | Trends in Anaesthesia and Critical Care (2021) | Populations did not fulfill. |
| 33 | Cost-effectiveness of the i-gel supraglottic airway device compared to tracheal intubation during out-of-hospital cardiac arrest: Findings from the AIRWAYS-2 randomised controlled trial | Stokes et al. | Resuscitation (2021) | Control group was not eligible for comparison with intervention groups. |
| 34 | Videolaryngoscopic versus direct laryngoscopic paraglossal intubation for cleft lip/palate reconstructive surgeries: A randomised controlled trial | Ray et al. | Indian Journal of Anaesthesia (2021) | Populations did not fulfill. |
| 35 | BOugie or stylet in patients UnderGoing Intubation Emergently (BOUGIE): Protocol and statistical analysis plan for a randomised clinical trial | Driver et al. | BMJ Open (2021) | Control group was not eligible for comparison with intervention groups. |
| 36 | Effects of direct laryngoscopy versus Glidescope videolaryngoscopy on subjective and objective measures of cognitive workload: an in-vivo randomized trial | Vuolato et al. | Minerva anestesiologica (2021) | Populations did not fulfill. |
| 37 | Comparison of C-MAC D-Blade with macintosh laryngoscope for endotracheal intubation in patients with cervical spine immobilization: A randomized controlled trial | Agrawal et al. | Trends in Anaesthesia and Critical Care (2021) | Populations did not fulfill. |
| 38 | Ease of intubation and incidence of dental injury during direct laryngoscopy: A randomized controlled trial comparing five different laryngoscope blades (Macintosh, Miller, Reduce flange, Blechman and Flangeless) | Mohanty et al. | Trends in Anaesthesia and Critical Care (2021) | Populations did not fulfill. |
| 39 | Fiberoptic bronchoscopy versus video laryngoscopy guided intubation in patients with craniovertebral junction instability: A cinefluroscopic comparison | Agrawal et al. | Surgical Neurology International (2021) | Populations did not fulfill. |
| 40 | Comparison of fibre-optic-guided endotracheal intubation through a supraglottic airway device versus hyperangulated video laryngoscopy by emergency physicians: A randomised controlled study in cadavers | Groombridge et al. | Hong Kong Journal of Emergency Medicine (2021) | Control group was not eligible for comparison with intervention groups. |
| 41 | Ventilatory performance of AMBU® AuraGain™ and LMA® Supreme™ in laparoscopic surgery: A randomised controlled trial | Zhang et al. | Anaesthesia and Intensive Care (2021) | Populations did not fulfill. |
| 42 | Intraocular pressure response to airway management: Comparison between LMA Supreme® and C-MAC® videolaryngoscope in day care surgery | Kaur et al. | Anaesthesia, Pain and Intensive Care (2020) | Populations did not fulfill. |
| 43 | Influence of head and neck positions on oropharyngeal seal pressure with Baska mask®versus I-gel™; A randomised clinical study | Sidhu et al. | Indian Journal of Anaesthesia (2020) | Populations did not fulfill. |
| 44 | Intubating Laryngeal Mask Airway-assisted Flexible Bronchoscopic Intubation Is Associated with Reduced Cervical Spine Motion When Compared with C-MAC Video Laryngoscopy-guided Intubation: A Prospective Randomized Cross over Trial | Swain et al. | Journal of Neurosurgical Anesthesiology (2020) | Populations did not fulfill. |
| 45 | Videolaryngoscopy versus direct laryngoscopy for double-lumen endotracheal tube intubation in thoracic surgery - A randomised controlled clinical trial | Risse et al. | BMC Anesthesiology (2020) | Populations did not fulfill. |
| 46 | King vision videolaryngoscopy versus direct laryngoscopy in patients requiring endotracheal intubation for general anaesthesia: A comparative study | Bashir et al. | JK Science (2020) | Populations did not fulfill. |
| 47 | Comparison of LM-Supreme™ and endotracheal tube in patients undergoing gynecological laparoscopic surgery | Kuvaki et al. | Journal of Clinical Monitoring and Computing (2020) | Populations did not fulfill. |
| 48 | Is video laryngoscopy easier than direct laryngoscopy for intubation in patients with contracture neck? | Gupta et al. | Saudi Journal of Anaesthesia (2020) | Populations did not fulfill. |
| 49 | Intubation using the gum-elastic bougie while wearing personal protective equipment | Milk et al. | American journal of disaster medicine (2020) | Populations did not fulfill. |
| 50 | Comparative study of the Ambu® AuraOnce™ laryngeal mask and endotracheal intubation in anesthesia airway management during neurosurgery | Zhang et al. | Journal of International Medical Research (2020) | Populations did not fulfill. |
| 51 | Comparison of the time to successful endotracheal intubation using the Macintosh laryngoscope or KingVision video laryngoscope in the emergency department: A prospective observational study | Mallick et al. | Turkish Journal of Emergency Medicine (2020) | This was not a suitable study design. |
| 52 | A randomised comparison of C-MAC™ and King Vision® videolaryngoscopes with direct laryngoscopy in 180 obstetric patients | Blajic et al. | International Journal of Obstetric Anesthesia (2019) | Populations did not fulfill. |
| 53 | GlideScope versus D-blade for tracheal intubation in cervical spine patients: A randomised controlled trial | Kumar et al. | Indian Journal of Anaesthesia (2019) | Populations did not fulfill |
| 54 | A comparison of blind intubation with the intubating laryngeal mask FASTRACH™ and the intubating laryngeal mask Ambu Aura-i™ a prospective randomised clinical trial | Schiewe et al. | BMC Anesthesiology (2019) | Populations did not fulfill. |
| 55 | I-Gel as an intubation conduit: Comparison of three different types of endotracheal tubes | Choudhary et al. | Indian Journal of Anaesthesia (2019) | Populations did not fulfill. |
| 56 | Video laryngoscopic intubation by emergency physicians: A comparison of endotracheal intubation between nonanaesthesiologist emergency physicians and anaesthesiologists | Sperber et al. | Notfall und Rettungsmedizin (2019) | Populations did not fulfill. |
| 57 | Video Laryngoscopy vs. Direct Laryngoscopy | Bakhsh et al. | Academic Emergency Medicine (2019) | This was not a suitable study design. |
| 58 | A single-centre, randomised controlled feasibility pilot trial comparing performance of direct laryngoscopy versus videolaryngoscopy for endotracheal intubation in surgical patients | Loughnan et al. | Pilot and Feasibility Studies (2019) | Populations did not fulfill. |
| 59 | Comparative evaluation of Ambu Aura-i and Fastrach™ intubating laryngeal mask airway for tracheal intubation: A randomized controlled trial | Anand et al. | Journal of Anaesthesiology Clinical Pharmacology (2019) | Control group was not eligible for comparison with intervention groups. |
| 60 | The Shikani Optical Stylet as an Alternative to Awake Fiberoptic Intubation in Patients at Risk of Secondary Cervical Spine Injury: A Randomized Controlled Trial | Mahrous et al. | Journal of Neurosurgical Anesthesiology (2018) | Control group was not eligible for comparison with intervention groups. |
| 61 | Tracheal intubation with channeled vs. Non-channeled videolaryngoscope blades | Biro et al. | Romanian Journal of Anaesthesia and Intensive Care (2018) | Populations did not fulfill. |
| 62 | A Randomised Controlled Trial Comparing Fibreoptic-Guided Tracheal Intubation through Two Supraglottic Devices: Ambu® Auragain™ Laryngeal Mask and LMA® Fastrach™ | Preece et al. | Anaesthesia and Intensive Care (2018) | Populations did not fulfill. |
| 63 | A prospective, randomized trial of the Ambu AuraGain™ laryngeal mask versus the LMA® protector airway in paralyzed, anesthetized adult men | Moser et al. | Minerva Anestesiologica (2018) | Populations did not fulfill. |
| 64 | Comparison of the effects of the endotracheal intubation with airtraq and macintosh laryngoscopes on hemodynamics | Acarel et al. | Gogus-Kalp-Damar Anestezi ve Yogun Bakim Dernegi Dergisi (2018) | Populations did not fulfill. |
| 65 | Usage of a semi-rigid intubation endoscope is not superior to a video laryngoscope. A prospective, randomised, controlled trial comparing the SensaScope vs. the McGrath Series 5 in surgical patients | Kriege et al. | Trends in Anaesthesia and Critical Care (2018) | Populations did not fulfill. |
| 66 | The usefulness of the McGrath MAC laryngoscope in comparison with Airwayscope and Macintosh laryngoscope during routine nasotracheal intubation: A randomaized controlled trial | Sato Boku et al. | BMC Anesthesiology (2017) | Control group was not eligible for comparison with intervention groups. |
| 67 | Flexible bronchoscopic intubation through the AuraGain™ laryngeal mask versus a slit Guedel tube: a non-inferiority randomized-controlled trial | Moser et al. | Canadian Journal of Anesthesia (2017) | Control group was not eligible for comparison with intervention groups. |
| 68 | Comparison of different stylets used for intubation with the C‐MAC D‐Blade® Videolaryngoscope: a randomized controlled study | Ömür et al. | Brazilian Journal of Anesthesiology (2017) | Populations did not fulfill. |
| 69 | Time-to-intubation in obese patients. A randomized study comparing direct laryngoscopy and videolaryngoscopy in experienced anesthetists | Ander et al. | Minerva Anestesiologica (2017) | Populations did not fulfill. |
| 70 | A novel technique for insertion of ProSeal™ laryngeal mask airway: Comparison of the stylet tool with the introducer tool in a prospective, randomised study | Myatra et al. | Indian Journal of Anaesthesia (2017) | Control group was not eligible for comparison with intervention groups. |
| 71 | Bougie assisted endotracheal intubation using the Air-Q™ Intubating Laryngeal Airway: A prospective randomized clinical study | Ebied et al. | Egyptian Journal of Anaesthesia (2017) | Control group was not eligible for comparison with intervention groups. |
| 72 | GlideScope versus McCoy laryngoscope: Intubation profile for cervically unstable patients in critical care setting | Ghanem et al. | Egyptian Journal of Anaesthesia (2017) | Populations did not fulfill. |
| 73 | A comparison of King Vision video laryngoscopy and direct laryngoscopy as performed by residents: a randomized controlled trial | Valencia et al. | Journal of Clinical Anesthesia (2016) | Populations did not fulfill. |
| 74 | Comparison of Cormack Lehane grading system and intubation difficulty score in patients intubated by D-blade video and direct Macintosh laryngoscope: A randomized controlled study | Pažur et al. | Acta Clinica Croatica (2016) | Populations did not fulfill. |
| 75 | A comparison between the GlideScope® classic and GlideScope® direct video laryngoscopes and direct laryngoscopy for nasotracheal intubation | Heuer et al. | Journal of Clinical Anesthesia (2016) | Populations did not fulfill. |
| 76 | Randomised comparison of the effectiveness of the laryngeal mask airway supreme, i-gel and current practice in the initial airway management of out of hospital cardiac arrest: A feasibility study | Benger et al. | British Journal of Anaesthesia (2016) | Populations did not fulfill. |
| 77 | Comparison of hemodynamic responses to laryngoscopy and intubation with Truview PCD™ , McGrath® and Macintosh laryngoscope in patients undergoing coronary artery bypass grafting: A randomized prospective study | Tempe et al. | Annals of Cardiac Anaesthesia (2016) | Populations did not fulfill. |
| 78 | Comparative effectiveness of McCoy laryngoscope and CMAC ® videolaryngoscope in simulated cervical spine injuries | Jain et al. | Journal of Anaesthesiology Clinical Pharmacology (2016) | Populations did not fulfill. |
| 79 | Intubation Success through I-Gel® and Intubating Laryngeal Mask Airway® Using Flexible Silicone Tubes: A Randomised Noninferiority Trial | Naik et al. | Anesthesiology Research and Practice (2016) | Populations did not fulfill. |
| 80 | The i-gel Supraglottic Airway as a Conduit for Fibreoptic Tracheal Intubation - A Randomized Comparison with the Single-use Intubating Laryngeal Mask Airway and CTrach Laryngeal Mask in Patients with Predicted Difficult Laryngoscopy | Michálek et al. | Prague medical report (2016) | Populations did not fulfill. |
| 81 | Randomised controlled trial comparing the Ambu® aScope 2 with a conventional fibreoptic bronchoscope in orotracheal intubation of anaesthetised adult patients | Chan et al. | Anaesthesia and Intensive Care (2015) | Populations did not fulfill. |
| 82 | Indirect videolaryngoscopy using Macintosh blades in patients with non-anticipated difficult airways results in significantly lower forces exerted on teeth relative to classic direct laryngoscopy: A randomized crossover trial | Pieters et al. | Minerva Anestesiologica (2015) | Populations did not fulfill. |
| 83 | Truview EVO2 laryngoscope reduces intubation difficulty in maxillofacial surgeries | Shrestha et al. | Journal of Oral and Maxillofacial Surgery (2015) | Populations did not fulfill. |
| 84 | A comparative study of tracheal intubation using i-gel™ and air-Q™ intubating LMA | Raza et al. | Anaesthesia, Pain and Intensive Care (2014) | Populations did not fulfill. |
| 85 | Glidescope videolaryngoscope vs Frova entrotracheal introducer in difficult unexpected airway management | Angeletti et al. | European Journal of Anaesthesiology (2014) | Populations did not fulfill. |
| 86 | Comparison of Aintree and Fastrach techniques for low-skill fibreoptic intubation in patients at risk of secondary cervical injury: A randomised controlled trial | Malcharek et al. | European Journal of Anaesthesiology (2014) | Populations did not fulfill. |
| 87 | A comparison of McCoy, TruView, and Macintosh laryngoscopes for tracheal intubation in patients with immobilized cervical spine | Bharti et al. | Saudi Journal of Anaesthesia (2014) | Populations did not fulfill. |
| 88 | Comparative study between Air-Q and Intubating Laryngeal Mask Airway when used as conduit for fiber-optic | Abdel-Halim et al. | Egyptian Journal of Anaesthesia (2014) | Populations did not fulfill. |
| 89 | Comparative study between LMA-Proseal™ and Air-Q® Blocker for ventilation in adult eye trauma patients | Youssef et al. | Egyptian Journal of Anaesthesia(2014) | Populations did not fulfill |
| 90 | Comparison of the efficacy of fiberoptic bronchoscopy and wireless video endoscope (disposcope®) in confirmation of the position of double lumen endotracheal tube | Kamburoʇlu et al. | Gogus-Kalp-Damar Anestezi ve Yogun Bakim Dernegi Dergisi(2014) | Population did not fulfill. |
| 91 | Prospective, randomized clinical trial of laryngeal mask airway Supreme(®) used in patients undergoing general anesthesia | Barreira et al. | Brazilian journal of anesthesiology (2013) | Population did not fulfill. |
| 92 | Efficacy of video laryngoscopy vs. direct laryngoscopy during urgent endotracheal intubation: A randomized controlled trial | Silverberg et al. | Chest (2013) | This was not a suitable study design. |
| 93 | Use of a video to improve patient and surrogate understanding of cardiopulmonary resuscitation and resuscitation preference options in the ICU: A randomized controlled trial | Wilson et al. | American Journal of Respiratory and Critical Care Medicine (2013) | This was not a suitable study design. |
| 94 | A comparison of Truview EVO2 laryngoscope with Macintosh laryngoscope in routine airway management: A randomized crossover clinical trial | Arora et al. | Saudi Journal of Anaesthesia (2013) | Population did not fulfill. |
| 95 | Indirect videolaryngoscopy with C-MAC D-Blade and GlideScope: A randomized, controlled comparison in patients with suspected difficult airways | Serocki et al. | Minerva Anestesiologica (2013) | Population did not fulfill. |
| 96 | Endotracheal intubation using videolaryngoscopy causes less cardiovascular response compared to classic direct laryngoscopy, incardiac patients according a standard hospital protocol | Maassen et al. | Acta Anaesthesiologica Belgica (2012) | Population did not fulfill. |
| 97 | A comparison of hemodynamic changes during laryngoscopy and endotracheal intubation by using three modalities of anesthesia induction | Lahsaee et al. | Anaesthesia, Pain and Intensive Care (2012) | Population did not fulfill. |
| 98 | Comparison of the air-Q ILA™ and the LMA-Fastrach™ in airway management during general anaesthesia | Neoh et al. | Southern African Journal of Anaesthesia and Analgesia (2012) | Population did not fulfill. |
| 99 | Comparative study between the use of Macintosh Laryngoscope and Airtraq in patients with cervical spine immobilization | Tolon et al. | Alexandria Journal of Medicine (2012) | Population did not fulfill. |
| 100 | Randomized controlled trial of endotracheal intubation using the c-mac videolaryngoscope versus standard laryngoscopy in patients undergoing emergent endotracheal intubation in the emergency department | Miner et al. | Academic Emergency Medicine (2012) | This was not a suitable study design. |
| 101 | Similar oropharyngeal leak pressures during anaesthesia with i-gel™, LMA-ProSeal™ and LMA-Supreme™ Laryngeal Masks | Van Zundert et al. | Acta Anaesthesiologica Belgica (2012) | Population did not fulfill. |
| 102 | GlideScope versus flexible fiber optic for awake upright laryngoscopy | Silverton | Annals of Emergency Medicine (2012) | Population did not fulfill. |
| 103 | GlideScope videolaryngoscope vs. Macintosh direct laryngoscope for intubation of morbidly obese patients: A randomized trial | Andersen et al. | Acta Anaesthesiologica Scandinavica (2011) | Population did not fulfill. |
| 104 | A randomised controlled trial comparing the McGrath® Videolaryngoscope with the straight blade laryngoscope when used in adult patients with potential difficult airways | Ng et al. | Anaesthesia and Intensive Care (2011) | Population did not fulfill. |
| 105 | Indirect versus direct laryngoscopy for routine nasotracheal intubation | Puchner et al. | Journal of Clinical Anesthesia (2011) | Population did not fulfill. |
| 106 | CobraPLUS and Cookgas air-Q versus Fastrach for blind endotracheal intubation: A randomised controlled trial | Erlacher et al. | European Journal of Anaesthesiology (2011) | Population did not fulfill. |
| 107 | A randomised, controlled crossover comparison of the C-MAC videolaryngoscope with direct laryngoscopy in 150 patients during routine induction of anaesthesia | Cavus et al. | BMC Anesthesiology (2011) | Population did not fulfill. |
| 108 | Comparison of the performance of 'Intubating LMA' and 'Cobra PLA' as an aid to blind endotracheal tube insertion in patients scheduled for elective surgery under general anesthesia | Darlong et al. | Acta Anaesthesiologica Taiwanica (2011) | Population did not fulfill. |
| 109 | Revisiting the value of pre-hospital tracheal intubation: An all time systematic literature review extracting the Utstein airway core variables | Lossius et al. | Critical Care (2011) | This was not a suitable study design. |
| 110 | Comparison of the Bonfils and Levitan optical stylets for tracheal intubation: A clinical study | Webb et al. | Anaesthesia and Intensive Care (2011) | Population did not fulfill. |
| 111 | Learning curves of the airtraq and the macintosh laryngoscopes for tracheal intubation by novice laryngoscopists: A clinical study | Di Marco et al. | Anesthesia and Analgesia (2011) | Population did not fulfill. |
| 112 | A comparison of the supreme™ laryngeal mask airway with the proseal™ laryngeal mask airway in anesthetized paralyzed adult patients: A randomized crossover study | Tham et al. | Canadian Journal of Anesthesia 2010 | Population did not fulfill. |
| 113 | Management of the predicted difficult airway: A comparison of conventional blade laryngoscopy with video-assisted blade laryngoscopy and the glidescope | Serocki et al. | European Journal of Anaesthesiology (2010) | Population did not fulfill. |
| 114 | Training resident anesthesiologists in adult challenging intubation comparing Truview EVO2™ and Macintosh laryngoscope: A preliminary study | Carlino et al. | Minerva Anestesiologica (2009) | Population did not fulfill. |
| 115 | Comparison of conditions for insertion of LMA - Proseal and LTS - II as alternatives for difficult intubation | Geprgiev et al. | Anaesthesiology and Intensive Care (2009) | Population did not fulfill. |
| 116 | Randomized controlled trial of the Pentax AWS®, Glidescope®, and Macintosh laryngoscopes in predicted difficult intubation | Malik et al. | British Journal of Anaesthesia (2009) | This was not a suitable study design. |
| 117 | Approach combining the airway scope and the bougie for minimizing movement of the cervical spine during endotracheal intubation | Takenaka et al. | Anesthesiology (2009) | Population did not fulfill. |
| 118 | Comparison of the Glidescope® and Pentax AWS® laryngoscopes to the Macintosh laryngoscope for use by Advanced Paramedics in easy and simulated difficult intubation | Nasim et al. | BMC Emergency Medicine (2009) | This was not a suitable study design. |
| 119 | The usefulness of the GlideScope video laryngoscope in the education of conventional tracheal intubation for the novice | You et al. | Emergency Medicine Journal (2009) | This was not a suitable study design. |
| 120 | Comparison of the intubating laryngeal mask airway with the bullard laryngoscope for endotracheal intubation in patients with simulated difficult airway using the philadelphia cervical collar | Khan et al. | Journal of Anaesthesiology Clinical Pharmacology (2009) | Population did not fulfill. |
| 121 | Evaluation of the Airtraq® and Macintosh laryngoscopes in patients at increased risk for difficult tracheal intubation | Maharaj et al. | Anaesthesia (2008) | Population did not fulfill. |
| 122 | Tracheal intubation of morbidly obese patients: A randomized trial comparing performance of Macintosh and Airtraq™ laryngoscopes | Ndoko et al. | British Journal of Anaesthesia (2008) | Population did not fulfill. |
| 123 | Tracheal intubation by non-anaesthetist physicians using the Airway Scope | Hirabayashi et al. | Emergency Medicine Journal (2007) | Population did not fulfill. |
| 124 | The intubating laryngeal mask: Is there a role for paramedics | Menzies et al. | Emergency Medicine Journal (2007) | Population did not fulfill. |
| 125 | Methods of endotracheal tube placement in patients undergoing pelviscopic surgery | Hwang et al. | Anaesthesia and Intensive Care (2007) | Population did not fulfill. |
| 126 | A comparison of the use of Trachlight® and Eschmann multiple-use introducer in simulated difficult intubation | Harvey et al. | European Journal of Anaesthesiology (2007) | This was not a suitable study design. |
| 127 | The ProSeal™ laryngeal mask airway is an effective alternative to laryngoscope-guided tracheal intubation for gynaecological laparoscopy | Lim et al. | Anaesthesia and Intensive Care (2007) | Control group was not eligible for comparison with intervention groups. |
| 128 | Comparison between classic laryngeal mask and cobra perilaryngeal airway during mechanical ventilation | Agah et al. | Tanaffos (2006) | Population did not fulfill. |
| 129 | A comparison of tracheal intubation using the Airtraq® or the Macintosh laryngoscope in routine airway management: A randomised, controlled clinical trial | Maharaj et al. | Anaesthesia (2006) | Population did not fulfill. |
| 130 | Tracheal intubation of morbidly obese patients: LMA CTrach™ vs direct laryngoscopy | Dhonneur et al. | British Journal of Anaesthesia (2006) | Population did not fulfill. |
| 131 | Comparison of the VBM™ laryngeal tube and laryngeal mask airway for ventilation during manual in-line neck stabilisation | Noor Zairul et al. | Singapore Medical Journal (2006) | Population did not fulfill. |
| 132 | Gum elastic bougie-guided insertion of the ProSeal™ laryngeal mask airway | Brimacombe et al. | Anaesthesia and Intensive Care (2004) | Population did not fulfill. |
| 133 | Paramedic-performed rapid sequence intubation of patients with severe head injuries | Ochs et al. | Annals of Emergency Medicine (2002) | Population did not fulfill. |
| 134 | Tracheal intubation and cervical spine excursion: Direct laryngoscopy vs. intubating laryngeal mask | Waltl et al. | Anaesthesia (2001) | Population did not fulfill. |
| 135 | Blind orotracheal intubation with the intubating laryngeal mask versus fibreoptic guided orotracheal intubation with the Ovassapian airway. A pilot study of awake patients | Dhar et al. | Anaesthesia and Intensive Care (2001) | Population did not fulfill. |
| 136 | Intubation through intubating laryngeal mask with and without a lightwand: A randomized comparison | Chan et al. | Anaesthesia and Intensive Care (2001) | Control group was not eligible for comparison with intervention groups. |
| 137 | Laryngeal mask vs intubating laryngeal mask: Insertion and ventillation by inexperienced resuscitators | Burgoyne et al. | Anaesthesia and Intensive Care (2001) | Population did not fulfill. |
| 138 | The oesophageal-tracheal Combitube Small Adult(TM) | Hartmann et al. | Anaesthesia (2000) | Population did not fulfill. |
| 139 | A comparison of blind and lightwand-guided tracheal intubation through the intubating laryngeal mask | Kihara et al. | Anaesthesia (2000) | Population did not fulfill. |
| 140 | Tracheal intubation with the Macintosh laryngoscope versus intubating laryngeal mask airway in adults with normal airways | Kihara et al. | Anaesthesia and Intensive Care (2000) | Population did not fulfill. |
| 141 | Complications following the use of the Combitube, tracheal tube and laryngeal mask airway | Oczenski et al. | Anaesthesia (1999) | Population did not fulfill. |
| 142 | Placement of the intubating laryngeal mask is easier than the laryngeal mask during manual in-line neck stabilization | Asai et al. | British Journal of Anaesthesia (1999) | Population did not fulfill. |
| 143 | Effect of the size of a tracheal tube and the efficacy of the use of the laryngeal mask for fibrescope-aided tracheal intubation | Koga et al. | Anaesthesia (1997) | Population did not fulfill. |
| 144 | Neuromuscular blockade-assisted oral intubation versus nasotracheal intubation in the prehospital care of injured patients | Rhee et al. | Annals of Emergency Medicine (1994) | Population did not fulfill. |
| 145 | Orotracheal intubation in patients with potential cervical spine injuries. An indication for the gum elastic bougie | Nolan et al. | Anaesthesia (1993) | Population did not fulfill. |
| 146 | Emergency intubation for acutely ill and injured patients | Lechy et al. | Cochrane Database of Systematic Reviews (2008) | This was not a suitable study design. |
| 147 | Bougie Use in Emergency Airway Management | - | - | This was not a suitable study design. |

**Table S4.** GRADE profile for assessing quality of evidence for the included studies for outcomes

| **Certainty assessment** | | | | | | | **№ of patients** | | **Effect** | | **Certainty** | **Importance** |
| --- | --- | --- | --- | --- | --- | --- | --- | --- | --- | --- | --- | --- |
| **№ of studies** | **Study design** | **Risk of bias** | **Inconsistency** | **Indirectness** | **Imprecision** | **Other considerations** | **videolaryngoscope** | **direct laryngoscope** | **Relative (95% CI)** | **Absolute (95% CI)** |  |  |
| **1. First success rate of endotracheal intubation (population after sensitivity analysis)** | | | | | | | | | | | | |
| 21 | randomised trials | not serious | very serious^a^ | not serious | not serious | none | 1520/1967 (77.3%) | 1470/1951 (75.3%) | **OR 1.36** (0.84 to 2.20) | **53 more per 1,000** (from 34 fewer to 117 more) | ⨁⨁◯◯ Low | CRITICAL |
| **2. First success rate of endotracheal intubation (intubation during CPR: less than 50%)** | | | | | | | | | | | | |
| 16 | randomised trials | not serious | very serious^a^ | not serious | not serious | none | 1118/1452 (77.0%) | 1078/1469 (73.4%) | **OR 1.52** (0.88 to 2.63) | **74 more per 1,000** (from 26 fewer to 145 more) | ⨁⨁◯◯ Low | CRITICAL |
| **2. First success rate of endotracheal intubation (intubation during CPR: 50% or more)** | | | | | | | | | | | | |
| 5 | randomised trials | not serious | very serious^a^ | not serious | not serious | none | 402/515 (78.1%) | 392/482 (81.3%) | **OR 0.99** (0.37 to 2.67) | **2 fewer per 1,000** (from 196 fewer to 108 more) | ⨁⨁◯◯ Low | CRITICAL |
| **3. First success rate of endotracheal intubation (country: non-Asian)** | | | | | | | | | | | | |
| 12 | randomised trials | not serious | very serious^a^ | not serious | not serious | none | 1023/1334 (76.7%) | 1043/1319 (79.1%) | **OR 1.01** (0.46 to 2.23) | **2 more per 1,000** (from 156 fewer to 103 more) | ⨁⨁◯◯ Low | CRITICAL |
| **3. First success rate of endotracheal intubation (country: Asian)** | | | | | | | | | | | | |
| 9 | randomised trials | not serious | very serious^a^ | not serious | not serious | none | 497/633 (78.5%) | 427/632 (67.6%) | **OR 1.84** (1.01 to 3.35) | **117 more per 1,000** (from 2 more to 199 more) | ⨁⨁◯◯ Low | CRITICAL |
| **4. First success rate of endotracheal intubation (difficulty airway: less than 50%)** | | | | | | | | | | | | |
| 13 | randomised trials | not serious | very serious^b^ | not serious | not serious | none | 790/1101 (71.8%) | 1224/1066 (114.8%) | **OR 0.80** (0.38 to 1.67) | **44 more per 1,000** (from 64 fewer to 366 more) | ⨁⨁◯◯ Low | CRITICAL |
| **4. First success rate of endotracheal intubation (difficulty airway: 50% or more)** | | | | | | | | | | | | |
| 2 | randomised trials | not serious | not serious | not serious | serious^c^ | none | 117/125 (93.6%) | 96/124 (77.4%) | **OR 4.27** (1.86 to 9.84) | **162 more per 1,000** (from 90 more to 197 more) | ⨁⨁⨁◯ Moderate | CRITICAL |
| **5. First success rate of endotracheal intubation (intubation experience: experienced)** | | | | | | | | | | | | |
| 10 | randomised trials | not serious | very serious^a^ | not serious | not serious | none | 745/945 (78.8%) | 746/923 (80.8%) | **OR 1.00** (0.33 to 3.09) | **0 fewer per 1,000** (from 226 fewer to 120 more) | ⨁⨁◯◯ Low | CRITICAL |
| **5. First success rate of endotracheal intubation (intubation experience: inexperienced)** | | | | | | | | | | | | |
| 11 | randomised trials | not serious | serious^d^ | not serious | not serious | none | 775/1022 (75.8%) | 724/1028 (70.4%) | **OR 1.54** (1.04 to 2.26) | **81 more per 1,000** (from 8 more to 139 more) | ⨁⨁⨁◯ Moderate | CRITICAL |
| **6. First success rate of endotracheal intubation (rapid sequence intubation: used)** | | | | | | | | | | | | |
| 15 | randomised trials | not serious | very serious^a^ | not serious | not serious | none | 1227/1592 (77.1%) | 1200/1588 (75.6%) | **OR 1.32** (0.73 to 2.40) | **48 more per 1,000** (from 63 fewer to 126 more) | ⨁⨁◯◯ Low | CRITICAL |
| **6. First success rate of endotracheal intubation (rapid sequence intubation: not used)** | | | | | | | | | | | | |
| 3 | randomised trials | not serious | very serious^a^ | not serious | not serious | none | 119/172 (69.2%) | 127/159 (79.9%) | **OR 0.66** (0.19 to 2.27) | **75 fewer per 1,000** (from 369 fewer to 101 more) | ⨁⨁◯◯ Low | CRITICAL |
| **7. First success rate of endotracheal intubation (study design: single-center)** | | | | | | | | | | | | |
| 18 | randomised trials | not serious | very serious^d^ | not serious | not serious | none | 1157/1472 (78.6%) | 1108/1485 (74.6%) | **OR 1.54** (0.88 to 2.70) | **73 more per 1,000** (from 25 fewer to 142 more) | ⨁⨁◯◯ Low | CRITICAL |
| **7. First success rate of endotracheal intubation (study design: multicenter)** | | | | | | | | | | | | |
| 3 | randomised trials | not serious | not serious | not serious | not serious | none | 363/495 (73.3%) | 362/466 (77.7%) | **OR 0.79** (0.58 to 1.06) | **44 fewer per 1,000** (from 108 fewer to 10 more) | ⨁⨁⨁⨁ High | CRITICAL |
| **8. First success rate of endotracheal intubation (setting: prehospital)** | | | | | | | | | | | | |
| 5 | randomised trials | not serious | very serious^a^ | not serious | not serious | none | 385/550 (70.0%) | 437/519 (84.2%) | **OR 0.38** (0.06 to 2.44) | **173 fewer per 1,000** (from 600 fewer to 87 more) | ⨁⨁◯◯ Low | CRITICAL |
| **8. First success rate of endotracheal intubation (setting: in-hospital)** | | | | | | | | | | | | |
| 16 | randomised trials | not serious | serious^d^ | not serious | not serious | none | 1135/1417 (80.1%) | 1033/1432 (72.1%) | **OR 1.86** (1.32 to 2.64) | **107 more per 1,000** (from 52 more to 151 more) | ⨁⨁⨁◯ Moderate | CRITICAL |

CI: confidence interval; OR: odds ratio; CPR: cardiopulmonary resuscitation

Explanations

a. The studies had substantial heterogeneity (I^2^>75%)

b. Neither the same direction nor a similar magnitude of the effect

c. Total sample size are too small

d. The studies had substantial heterogeneity.

**Figure S1.** Type of laryngoscopes


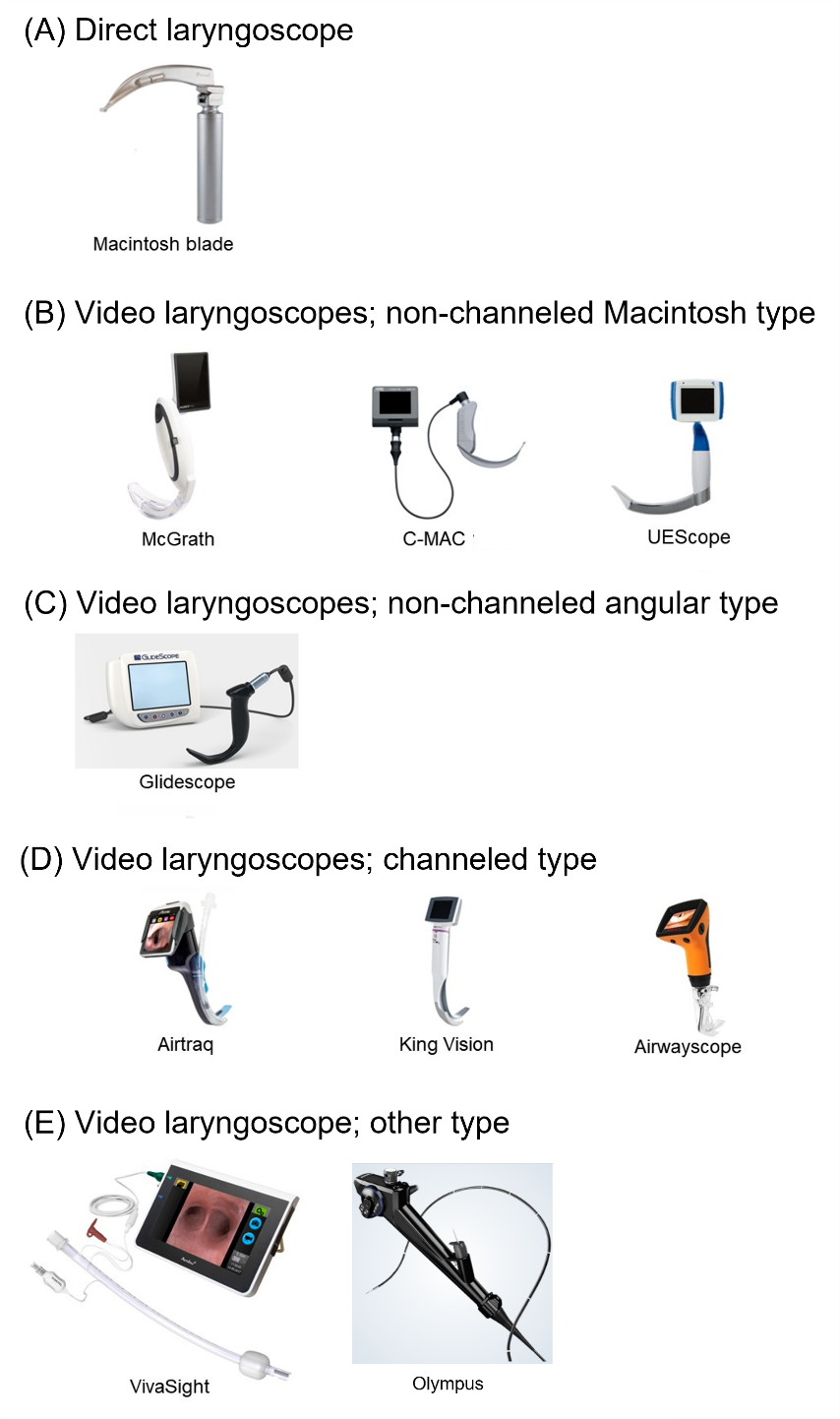
`

(A) Direct laryngoscope with Macintosh blade. (B) Video laryngoscopes; non-channeled Macintosh type A.P.Advance® (available online: <https://www.red-dot.org/project/venner-ap-advance-video-laryngoscope-10436>) and McGrath® (available online: <https://www.medtronic.com/>) (C) Video laryngoscopes; non-channeled angular type. Glidescope® (available online: <https://www.verathon.com/>), C-MAC with D-blade® (available online: <https://www.karlstorz.com/>), and UEScope® (available online: <https://www.uescope.com/>) (D) Video laryngoscopes; channeled type. Airtraq® (available online: <https://www.airtraq.com/>), King Vision (available online: <https://www.ambuusa.com/>), and Airwayscope® (available online: <https://medical.crkennedy.com.au/>) (E) Video laryngoscope; other type. VivaSight® (available online: <https://www.ambu.com/>) and Olympus® Bronchoscope (available online: <https://medical.olympusamerica.com/>)

**Figure S2.** Graph of quality assessment of included studies


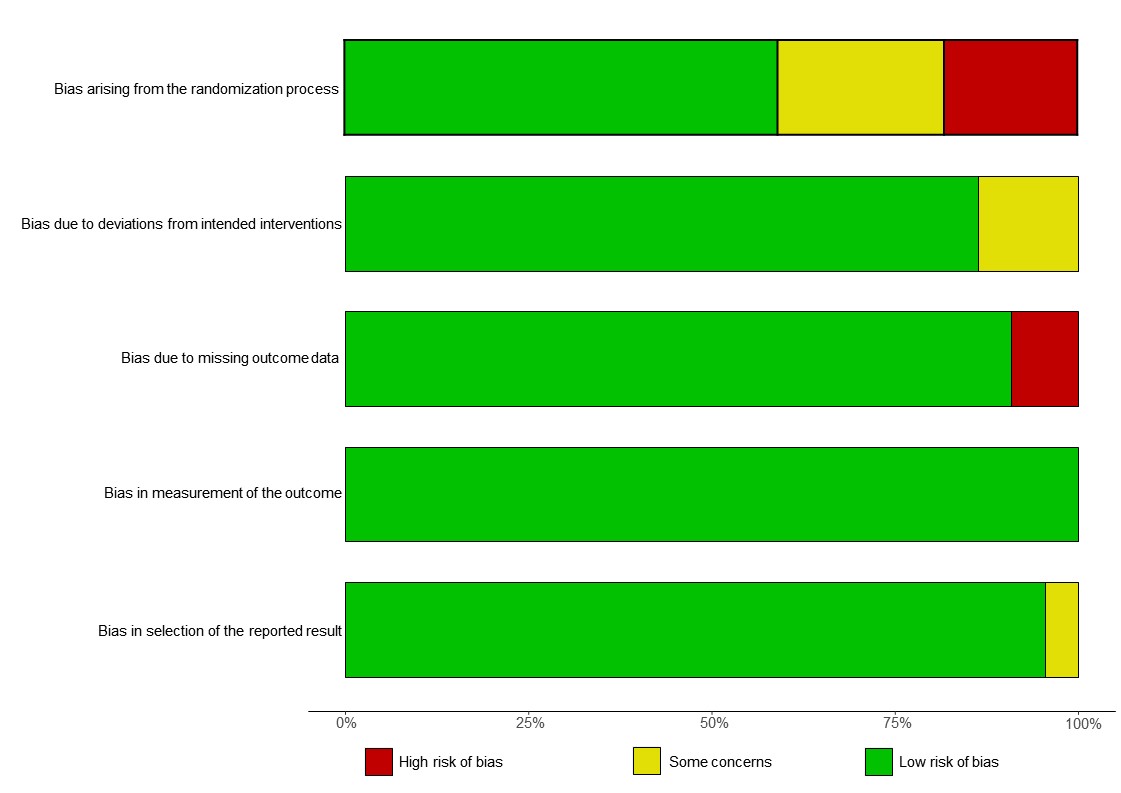


(A) Bar graph for each domain of quality assessment


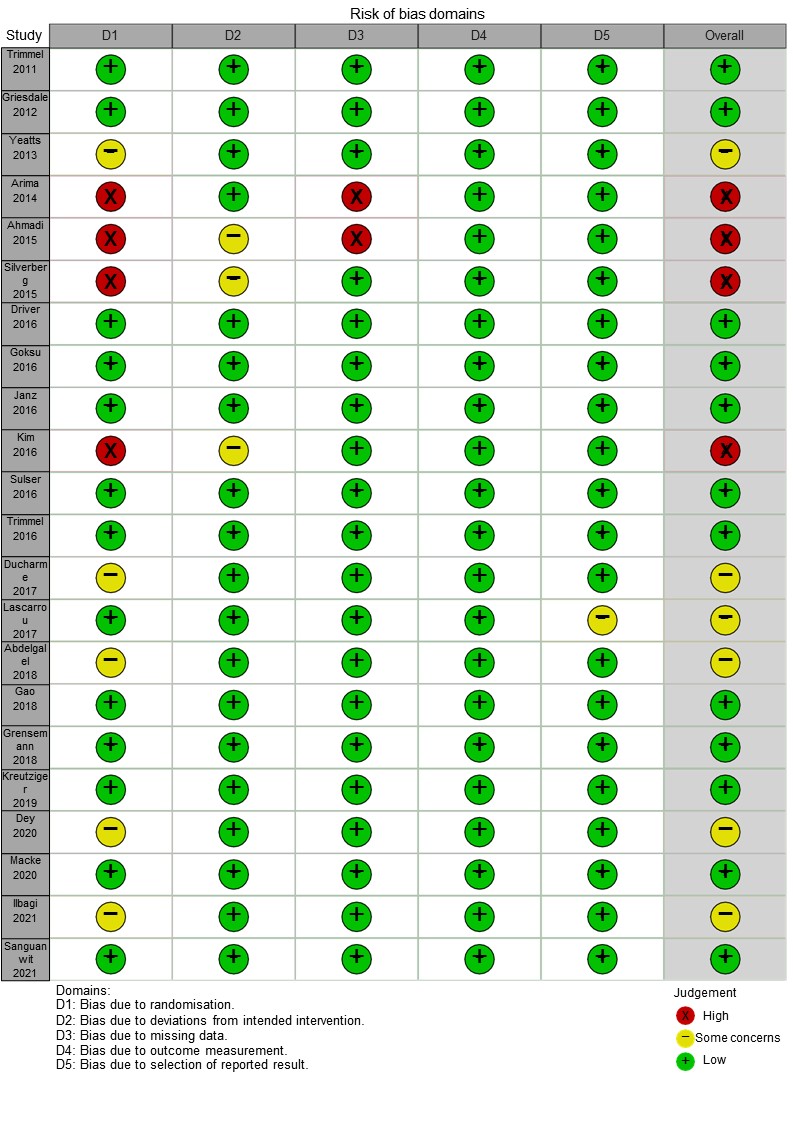


(B) Details in quality assessment for each study.

**Figure S3.** Funnel plot assessing publication bias for outcome

**
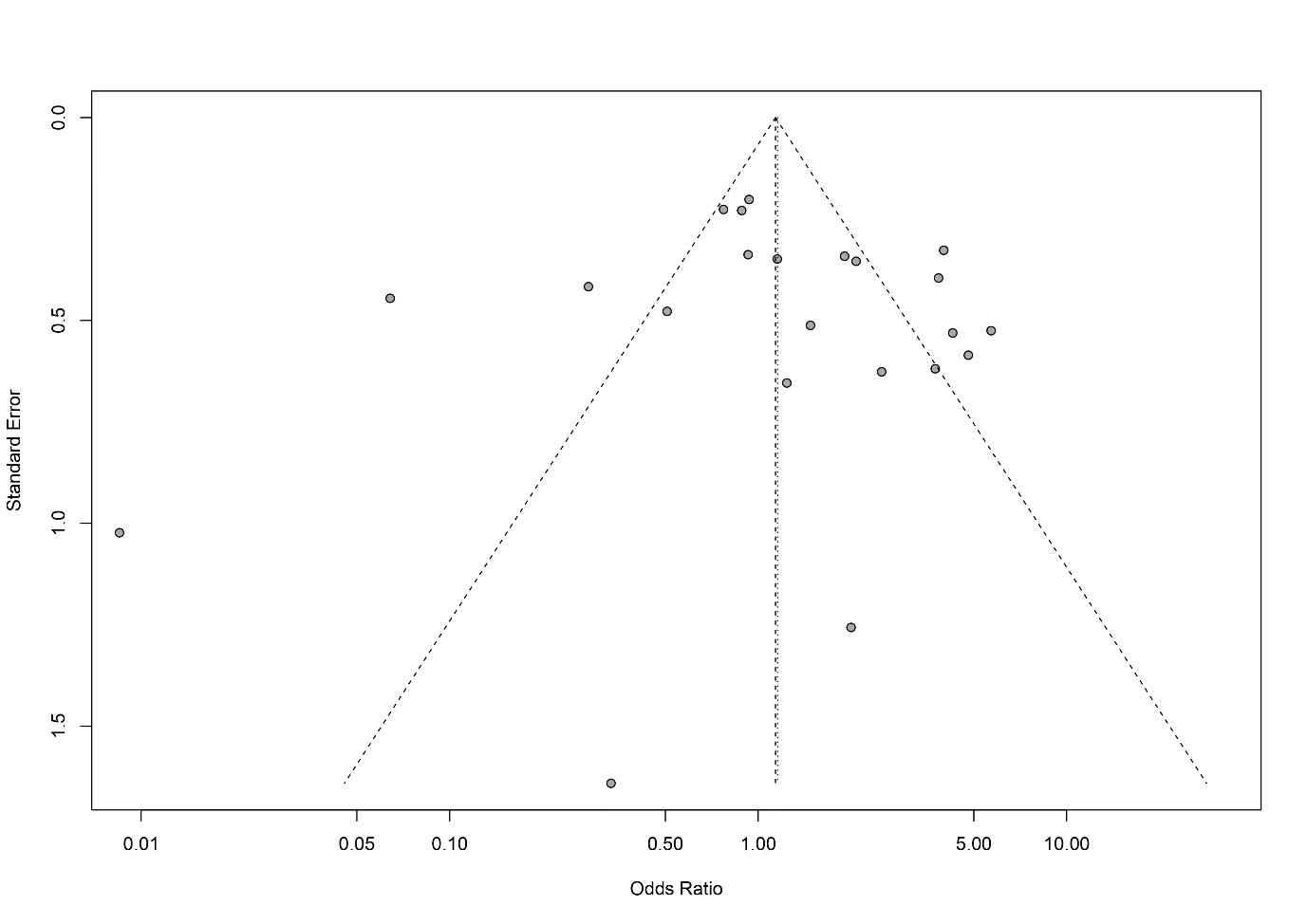
**

**Figure S4.** Forest plot and heterogeneity plot for first attempt success between direct and video laryngoscope

**
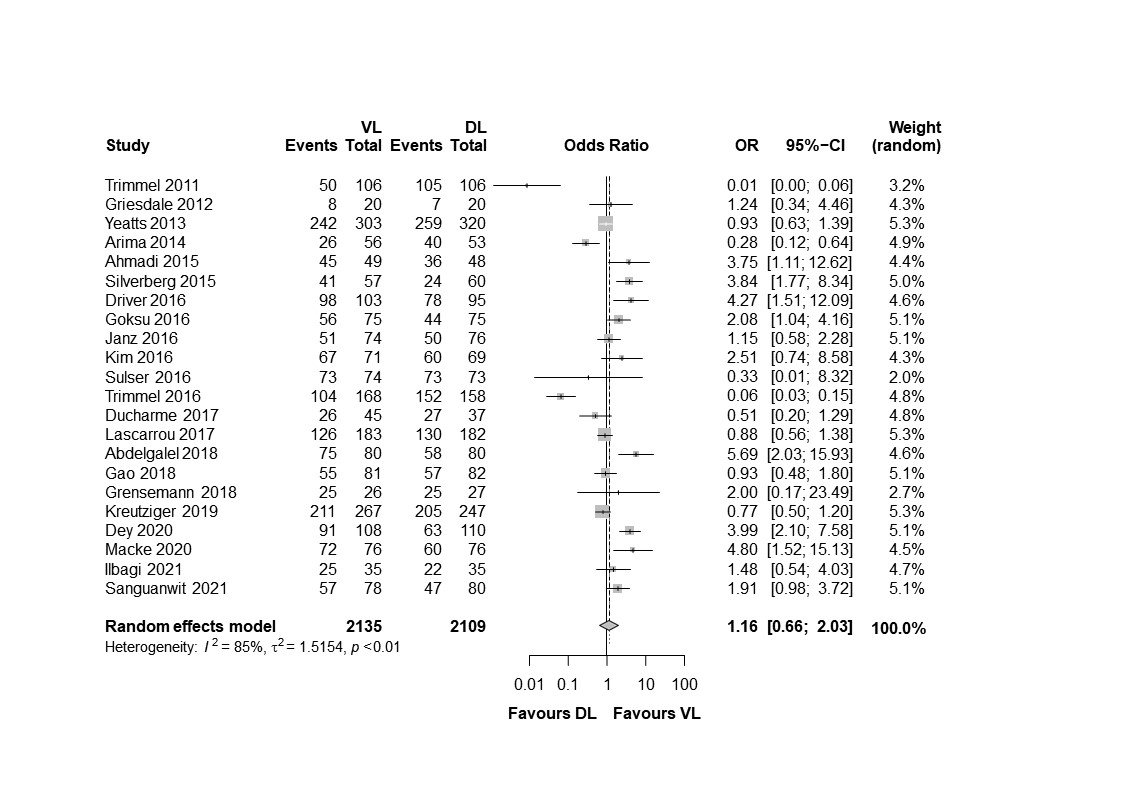
**

(A) Forest plot of total studies.


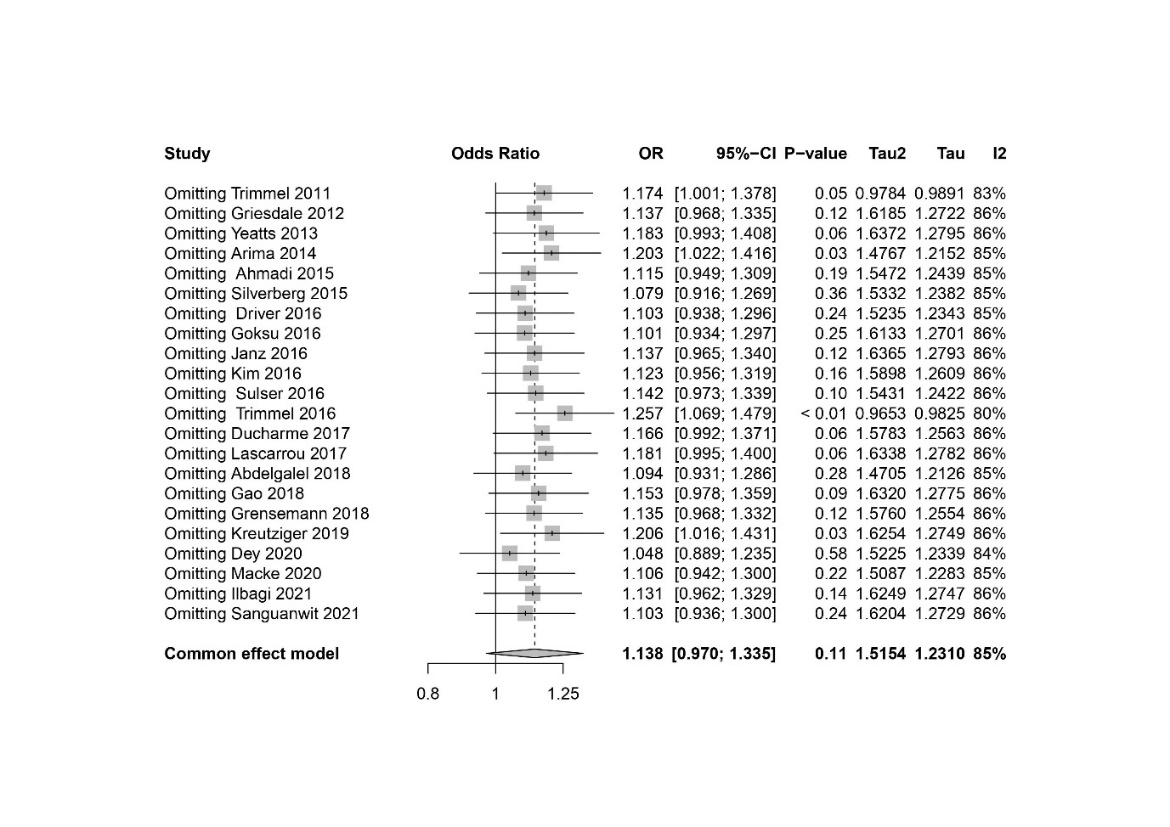

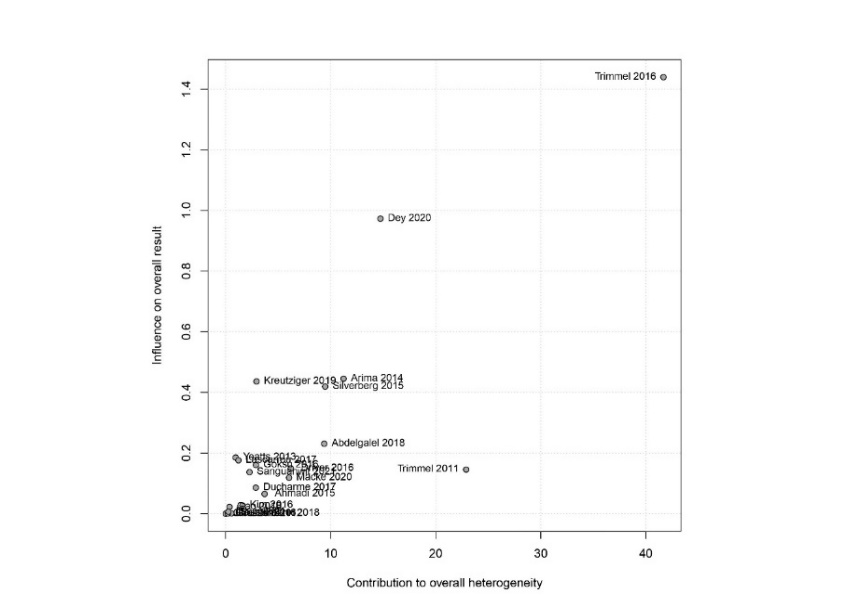


Baujat Plot

(B) Sensitivity analysis to identify outlier study. Sensitivity analysis revealed that excluding Trimmel's 2016 study significantly reduced the heterogeneity of pooled results (up to I^2^ 80%, p-value < 0.01). Trimmel's 2016 study also contributed the most heterogeneity, according to the Baujat plot. These findings demonstrated that Trimmel's 2016 study was a significant outlier.


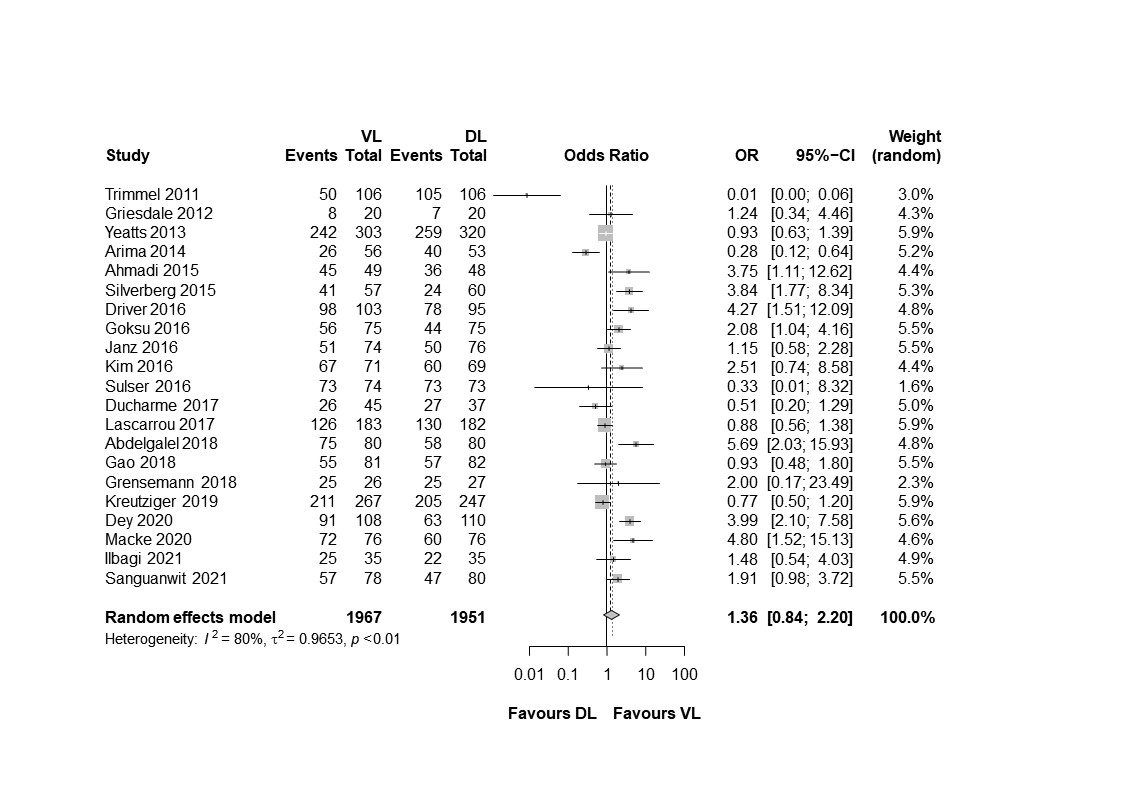


(C) Forest plot of 21 studies after sensitivity analysis. Trimmel's 2016 study was removed because sensitivity analysis revealed it to be an outlier.

**Figure S5.** Forest plot in subgroup analysis for all factors except study design and difficult airway

**
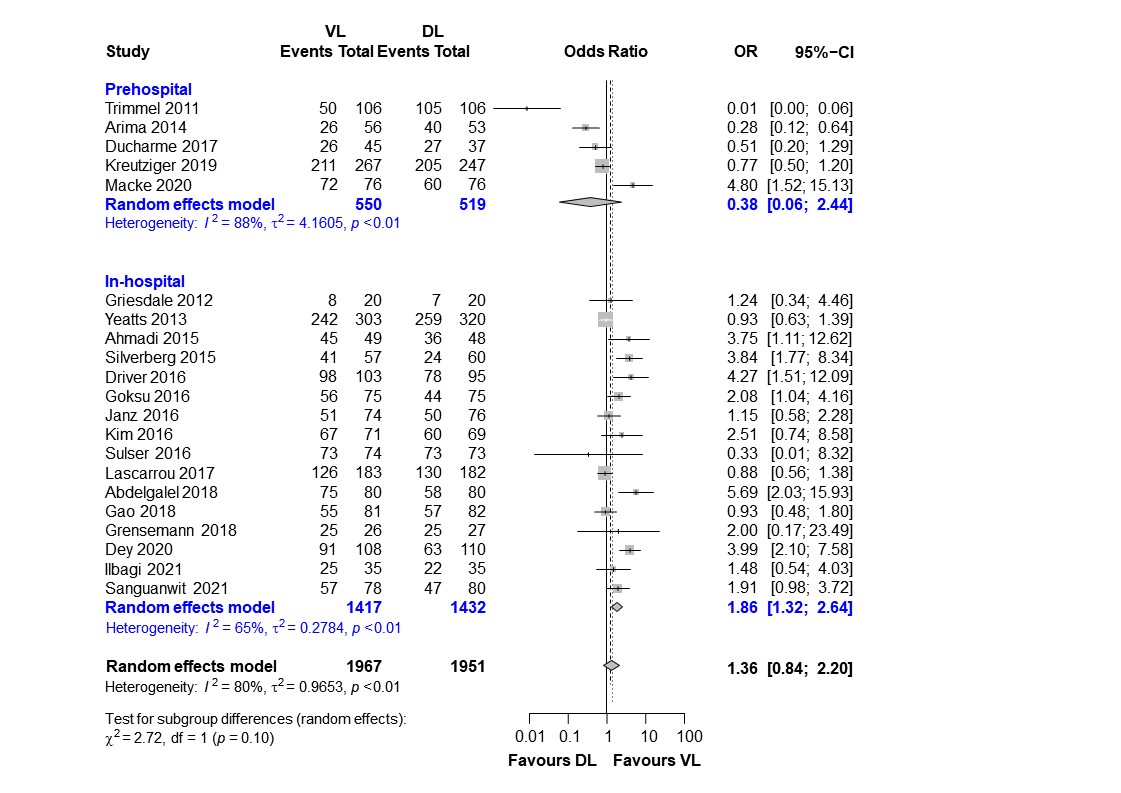
**

(A) Prehospital vs. In-hospital setting

**
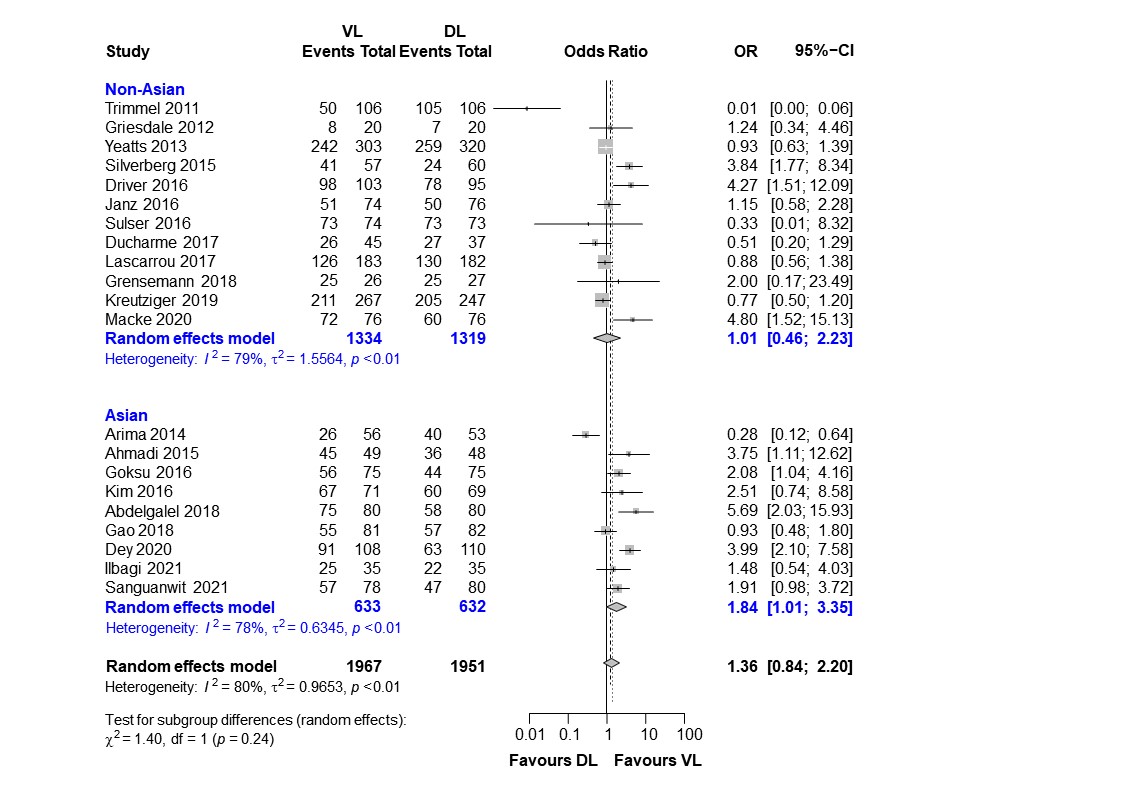
**

(B) Non-Asian vs. Asian countries

**
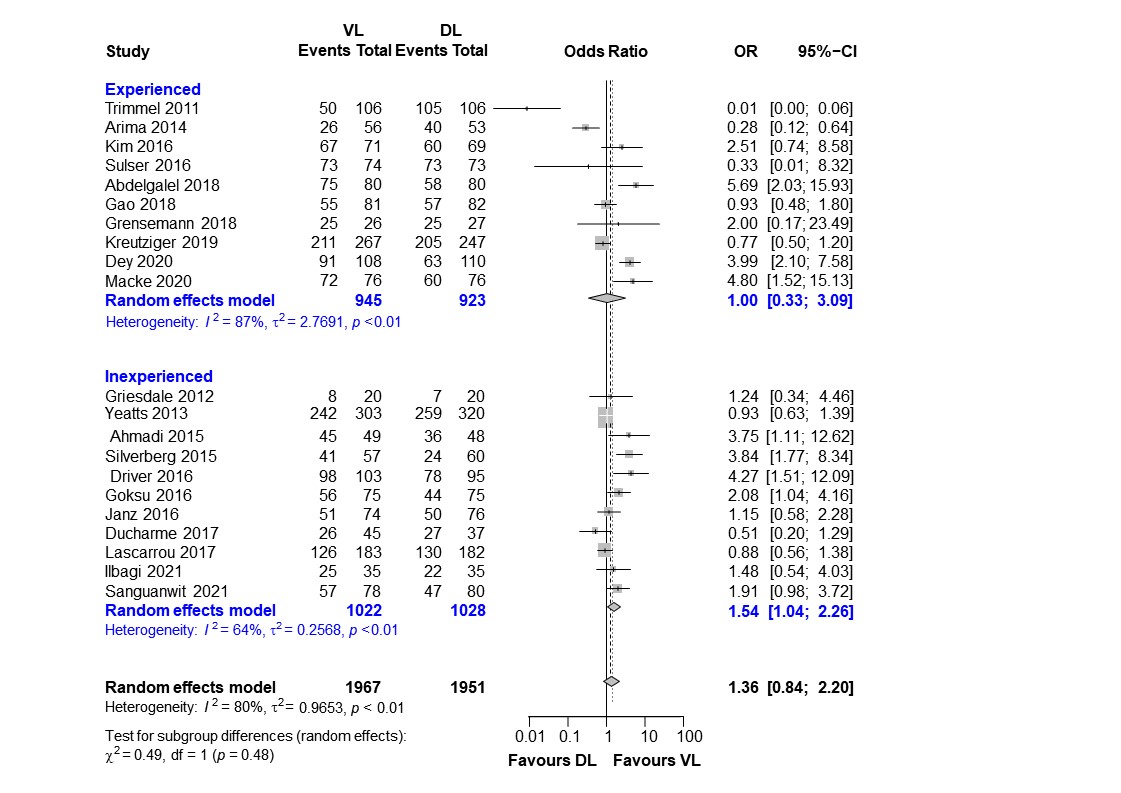
**

(C) Inexperienced vs. Experienced practitioners for endotracheal intubation

**
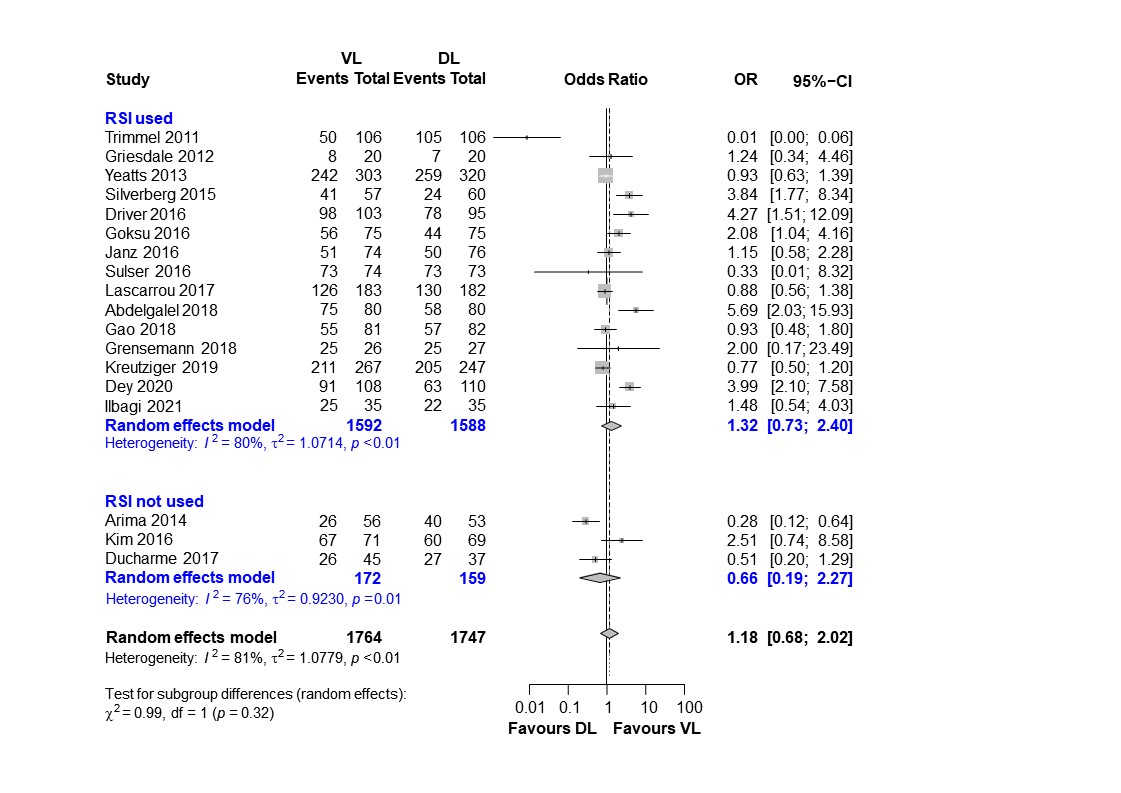
**

(D) Rapid sequence intubation (RSI) vs. Non-RSI

**
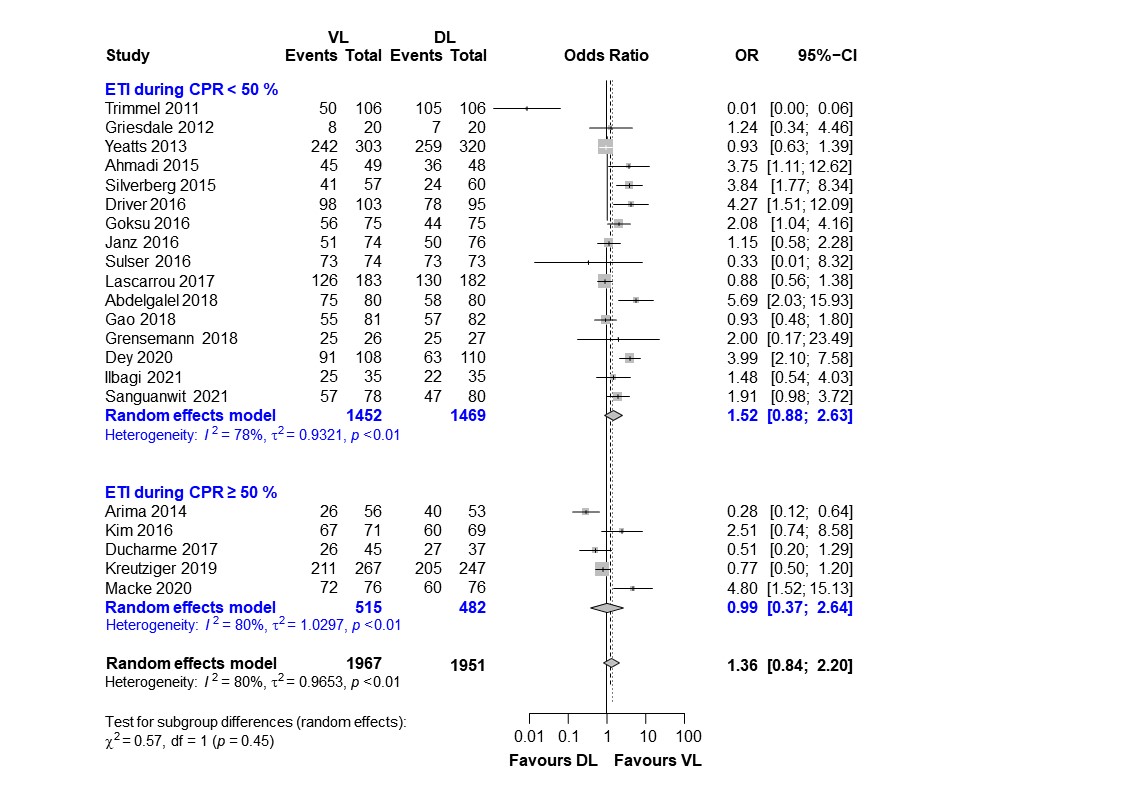
**

(E) Less than 50% vs. 50% or more of intubation during the cardiopulmonary resuscitation
